# Phylogenetic Distribution of Extant Richness Suggests Metamorphosis Is a Key Innovation Driving Diversification in Insects

**DOI:** 10.1371/journal.pone.0109085

**Published:** 2014-10-02

**Authors:** James L. Rainford, Michael Hofreiter, David B. Nicholson, Peter J. Mayhew

**Affiliations:** 1 Department of Biology, University of York, York, United Kingdom; 2 Faculty of Mathematics and Natural Sciences, Institute for Biochemistry and Biology, University of Potsdam, Potsdam, Germany; 3 Department of Natural Sciences, National Museums Scotland, Edinburgh, United Kingdom; 4 Department of Earth Sciences, The Natural History Museum, London, United Kingdom; BiK-F Biodiversity and Climate Research Center, Germany

## Abstract

Insects and their six-legged relatives (Hexapoda) comprise more than half of all described species and dominate terrestrial and freshwater ecosystems. Understanding the macroevolutionary processes generating this richness requires a historical perspective, but the fossil record of hexapods is patchy and incomplete. Dated molecular phylogenies provide an alternative perspective on divergence times and have been combined with birth-death models to infer patterns of diversification across a range of taxonomic groups. Here we generate a dated phylogeny of hexapod families, based on previously published sequence data and literature derived constraints, in order to identify the broad pattern of macroevolutionary changes responsible for the composition of the extant hexapod fauna. The most prominent increase in diversification identified is associated with the origin of complete metamorphosis, confirming this as a key innovation in promoting insect diversity. Subsequent reductions are recovered for several groups previously identified as having a higher fossil diversity during the Mesozoic. In addition, a number of recently derived taxa are found to have radiated following the development of flowering plant (angiosperm) floras during the mid-Cretaceous. These results reveal that the composition of the modern hexapod fauna is a product of a key developmental innovation, combined with multiple and varied evolutionary responses to environmental changes from the mid Cretaceous floral transition onward.

## Introduction

Hexapoda, including insects and their six-legged relatives, are the most species-rich animal clade in terrestrial ecosystems and collectively comprise over half of all described extant species [1], [2]. Therefore understanding the origins of this exceptional richness is key to understanding the history of life on land and the assembly of terrestrial ecosystems [3]. In addition to their high overall species richness, insect groups are also remarkable for the degree of disparity in richness existing among the major sub-clades. For example the orders Zoraptera (“angel insects”) and Coleoptera (beetles) differ in richness by four orders of magnitude (32 and 350,000 described extant species, respectively [2]). A key part of the discussion on these differences in extant richness relates to the hypothesized effects of potential key innovations that may have acted as drivers for hexapod richness [3]. Such proposed innovations include both major morphological developments including the origin of the insect body plan [2]–[4], flight [2]–[6], the capacity to fold the wings [7], [8] and the origin of complete metamorphosis [2]–[4], [9], and ecological opportunities or innovations, notably the evolution of flowering plants (angiosperms) [10]–[12] and parasitism [13].

Attempts to explicitly test these ideas within a phylogenetic framework have either been restricted to particular orders [14]–[16], thus omitting a wider context, or have ignored variation within orders [7], [8]. Here we integrate these disparate approaches by producing a dated hypothesis of phylogenetic relationships across the hexapods that is near-complete at the family level, through the combination of previously published molecular sequence data and a set of literature derived constraints (see below and Supplementary materials). Our goal is therefore not to present a novel estimate of the hexapod phylogeny (see discussion below), but instead to focus on what current taxonomic, phylogenetic and paleontological evidence reveals about broad patterns of diversification within the group, and its relationship with key evolutionary innovations, environmental changes and mass extinctions [17]–[19].

## Results

**Figure 1:**
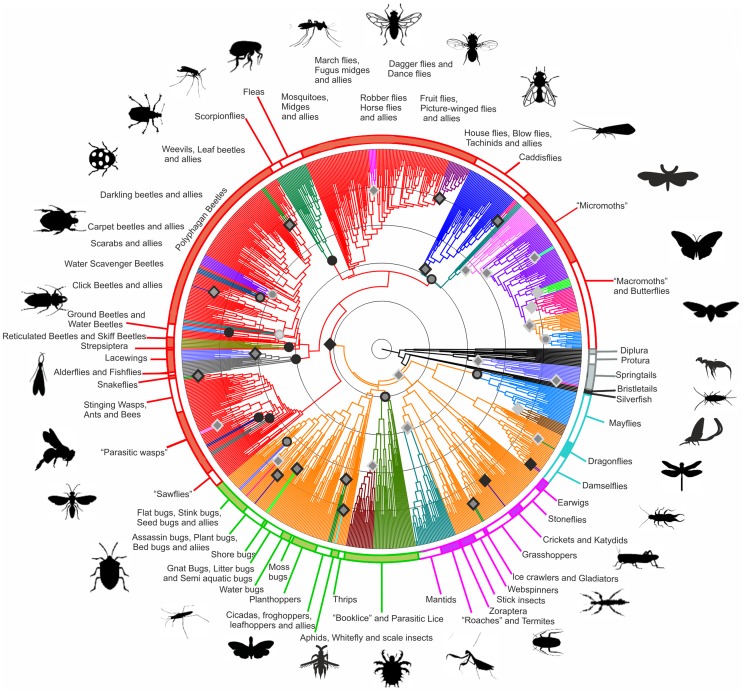
Dated phylogeny of extant hexapod families showing diversification rate shifts. The tree shown is from a maximum likelihood analysis of 8 genes, calibrated by 89 fossils. Membership of major clades is denoted by coloration of the ring (grey: Entognatha, black: basal insects, cyan: Palaeoptera, magenta: Polyneoptera, green: Paraneoptera, red: Holometabola). Changes in branch coloration denote diversification shifts identified using TurboMEDUSA (Table S3). Branch colors identify regions of the tree with the same underlying diversification model. Symbols at shifts denote a net upshift (diamond) or down shift (circle). Coloration of symbols reflects the robustness of the shift event across 500-scaled samples taken from the post-burin MCMC chain (black: shift recovered in >80% of samples, grey with black outline: recovery >50%, grey with pale outline: recovery >30%, pale grey: recovery<30%). Black rings are shown at 100 Ma increments from the present. See Supplementary materials for further details and discussion. See also Figures S1–S3, Tables S1–S4, and Datafiles S1, S2.

The dated phylogeny used in this study contains 874 higher taxa of Hexapoda (Fig. 1). Taxa were variously resolved to family or superfamily level, such that the presented tree incorporates a total of 903 of the approximately 1100 recognized extant families, with taxonomy following that given by GenBank references up to August 2013 (see Supplementary materials for further discussion). The tree was reconstructed using a combination of eight widely sampled molecular markers and literature-derived constraints on certain widely recognized phylogenetic nodes ([20], [21] see Supplementary materials for details). The tree topology was inferred using a partitioned RAxML (maximum likelihood) analysis [22], [23]. This topology was dated using a relaxed molecular clock implemented in MrBayes 3.2 [24] and calibrated using 86 fossil dates taken from the recent palaeoentomological literature (Table S2).

Using our dated tree we estimated the crown divergence of Hexapoda, i.e. the divergence of true insects from Entognatha (basal hexapods including springtails) as occurring in the Ordovician (mean estimate 474.4 Ma, 95% CI 439.6–502.9 Ma), which is consistent with other recent molecular clock estimates [25]–[27] (Fig S2). These estimates greatly exceed the age of the oldest securely placed hexapod fossils including the potential crown winged insect *Rhyniognatha hirsti* from the early Devonian [28]. Little is known regarding Devonian insect communities [2] and the nature of terrestrial communities at this early date remains poorly understood [29]. However, our results are in line with recent fossil evidence indicating an early (i.e. prior to the late Carboniferous) origin for major crown lineages, including the stem lineages of several orders of advanced Holometabola (insects that undergo complete metamorphosis) [30], [31].

**Figure 2:**
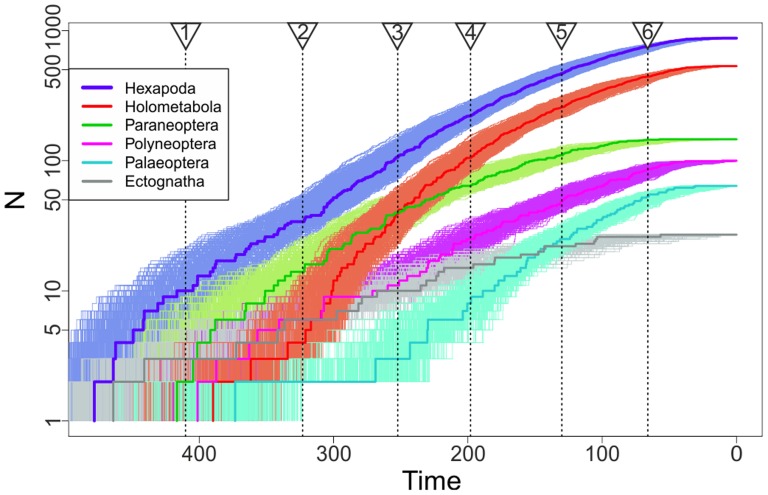
Lineage (y-axis; log scale) through time (x-axis; Ma) plot for the major groups of Hexapoda using the phylogeny in Fig. 1. Colors used identify the same clades as the ring in Fig. 1. Thick lines are calculated from the mean tree dates (Fig. 1). Shaded regions represent 500-scaled samples taken from the MCMC chain used in dating. Major events in the history of the group are denoted using dotted lines: 1. Oldest Hexapod fossil. 2. Oldest member of crown Pterygota (Polyneoptera). 3. Permo-Triassic mass extinction. 4. Origination of crown Angiosperms [44]. 5. Angiosperms become abundant in fossil record. 6. Cretaceous-Paleocene mass extinction.

At higher taxonomic levels, lineage through time plots (Fig. 2) indicate a remarkable stability in divergence rate across all the major hexapod clades, with some suggestion of an elevated diversification rate in Holometabola during the late Permian corresponding to basal divergences within Coleoptera and Diptera (flies) [2], [32]. Despite the conventional division between Paleozoic and post-Paleozoic insect faunas in paleoentomological research [2], [33], our results reveal no evidence for changes in diversification rate around the time of the Permo-Triassic extinction event (P/T) (Fig. 2), suggesting that the radiation of extant groups was not strongly impacted by the loss of Paleozoic forms indicated by the fossil record [17], [18]. A possible exception is an upshift in the diversity of Palaeoptera (dragonflies and mayflies) associated with the origin of crown members of the two orders, both of which undergo major taxonomic turnover during the P/T event [2], [34] (Fig. 2).

Despite this apparent stability in the origination of higher taxa, the application of birth-death models [35], [36] identifies two major transitions, characterized as shifts in the net diversification rate and turnover in the descendent clades, which together play a major role in defining the overall structure of hexapod diversification. These major shifts correspond to the origins of flight (Pterygota) and of complete metamorphosis (Holometabola) (Fig. 1, Table 1 and Table S3). Both in terms of the degree to which its inclusion improves the likelihood of diversification models (Table 1) and in its relative stability with respect to uncertainties in node age estimation (Fig. 1, Table S3), the upshift in diversification rate associated with the origin of complete metamorphosis represents the more strongly supported event. Previous studies proposing a link between complete metamorphosis and elevated diversification rates have been based on evidence in the fossil record [9], [37], which for hexapods is highly incomplete [38]. In contrast, sister group comparisons, using earlier phylogenetic reconstructions [20], [21], failed to recover a diversification shift associated with Holometabola [7], [8]. However, likelihood ratio tests indicate that the birth-death models significantly favor this position over alternative proposals including Eumetabola (Holometabola plus its sister group) and Neoptera (insects able to fold their wings; Table 1). Earlier studies [8], [39] have also provided some evidence supporting the role of flight in promoting hexapod diversification. Although our analysis supports this notion it also shows that the recovery of this shift is sensitive to uncertainties in divergence time estimates within the phylogeny rendering its overall role in hexapod diversification ambiguous (Fig. 1).

**Table 1:** Log likelihood and parameter estimates for models with a single shift in diversification rate.

Taxon 1	*lnL*	R root	R clade	Taxon 2	*lnL*	R root	R clade	ChiSquared	DF	p.value
Holometabola	−11299.630	0.00359686	0.0112969	**Uniform Model**	−11504.581	0.0087352	-	409.90	3	<0.001
Holometabola	−11299.630	0.00359686	0.0112969	**Obtectomera (Second Best Node)**	−11351.037	0.0073964	0.0264802	102.81	1	<0.001
Holometabola	−11299.630	0.00359686	0.0112969	**Paraneoptera**	−11433.738	0.0096620	0.0039822	268.22	1	<0.001
Holometabola	−11299.630	0.00359686	0.0112969	**Eumetabola**	−11393.178	0.0032761	0.0098645	187.10	1	<0.001
Holometabola	−11299.630	0.00359686	0.0112969	**Neoptera**	−11415.252	0.0020526	0.0093177	231.25	1	<0.001
Holometabola	−11299.630	0.00359686	0.0112969	**Pterygota**	−11474.292	0.0023812	0.0089912	349.32	1	<0.001
Holometabola	−11299.630	0.00359686	0.0112969	**Insecta**	−11481.919	0.0026486	0.0089487	364.58	1	<0.001
Holometabola	−11299.630	0.00359686	0.0112969	**Coleoptera**	−11495.184	0.0082814	0.0105405	391.11	1	<0.001
Holometabola	−11299.630	0.00359686	0.0112969	**Lepidoptera**	−11418.241	0.0075482	0.016791	237.22	1	<0.001
Holometabola	−11299.630	0.00359686	0.0112969	**Diptera**	−11486.288	0.0082253	0.0120813	373.32	1	<0.001
Holometabola	−11299.630	0.00359686	0.0112969	**Hymenoptera**	−11497.610	0.0085002	0.0111554	395.96	1	<0.001

Log likelihood tests performed to compare the optimal model, with shift placed on Holometabola, and various alternative models. Data shown are log likelihoods of the respective models estimated in laser using turnover estimates as estimated in the homogenous model in MEDUSA (i.e. that with no shifts). Net diversification rates are estimated for the partition including the root (R_root_) and the descendants of the focal node (R_clade_). Chi squared values, degrees of freedom and p values relate to results of likelihood ratio comparisons between the denoted models. See also Table S3.

In addition to these broad patterns, diversification shift models identified a further forty-three clades on the tree potentially associated with shifts in diversification rates (Fig. 1, Table S3). These shifts vary in their intensity and robustness with respect to uncertainties in branch length and are distributed across the tree, with the majority occurring within the holometabolan radiation. Among the most robust and phylogenetically inclusive shifts are down-shifts impacting on known or suspected relict groups within the modern fauna. These include holometabolan groups such as Neuropterida (lacewings and their relatives), Mecoptera and Siphonaptera (scorpionflies and fleas [40]) and basal members of Coleoptera (beetles) and Lepidoptera (moths), as well as non-metamorphosing groups such as Ephemeroptera (mayflies) and Psocodea (booklice and parasitic lice) [41]. The fossil records for a number of these groups indicate a higher family richness during the Mesozoic, suggesting that their current representatives are surviving relics of taxa that were formerly more diverse [17], [18], further supporting the results of the diversification shift models.

**Figure 3:**
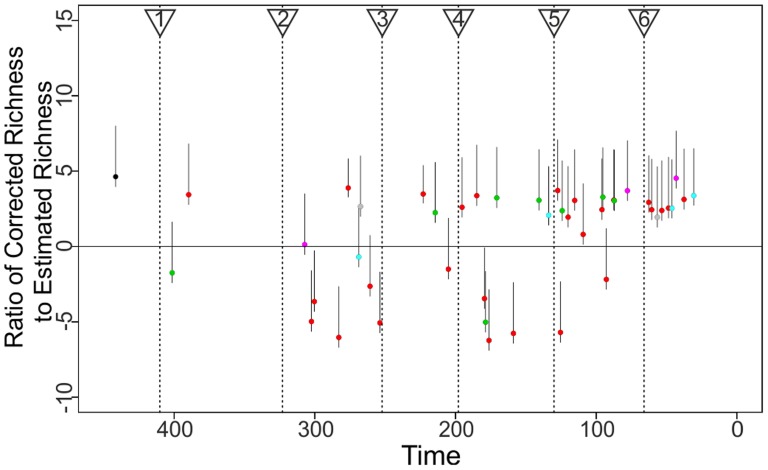
Change in species richness associated with shift events plotted through time. Values plotted show the ratios between the observed richness of the clade (after correction for nested shifts) and the mean estimated values of the richness of a clade of the appropriate age under the parental diversification model (see main text and Supplementary materials). Confidence intervals given are based on the change in richness associated with 95% CIs on the estimated outcomes of the stochastic diversification process. See also Table S3.

In contrast with these relict groups, most of the shifts leading to a net increase in taxonomic richness are comparatively recent (Fig. 3) and are associated with restricted, but massively diverse lineages many of which are of large ecological significance in recent communities. Among the non-holometabolan insects these include large herbivorous radiations such as the katydids (Tettigoniidae), true and lubber grasshoppers (Acrididae and Romaleidae), aphids (Aphidoidea), leafhoppers and treehoppers (Membracoidea), as well as plant/lace bugs and stink bugs (Miridae/Tingidae and Pentatomidae). Also represented are predatory groups such as assassin bugs (Reduviidae) and certain families of dragonflies and damselflies (Odonata). The pattern of shifts within the Dictyoptera (which includes detritivorous roaches and termites as well as predatory mantids) [42] is unstable with respect to branch length, with the majority of samples failing to recover the small proposed shift encompassing the entirety of the group (Fig. S3, Table S4). These groups, with the exception of Dictyoptera, radiated during the mid to late Cretaceous, which may imply an association between these radiations and the restructuring of floral and faunal communities during this interval following the radiation of angiosperms [43], [44].

Unsurprisingly, several upshifts in diversification within Holometabola also involve groups directly associated with the angiosperm radiation, with notable examples including leaf and longhorn beetles (Chrysomeloidea) [15], [45] and advanced bees (Apidae and Megachilidae) [46]. Our results also strongly support an upshift encompassing the Calyptratae, which includes houseflies and the important parasitoid group Tachinidae [14]. The recovered pattern of diversification shifts in Lepidoptera is complex and highly sensitive to uncertainties in branch length estimation, reflecting the difficulties of accurately dating a group for which there is a lack of suitable calibration fossils [17], [47], and which includes several regions of phylogenetic instability [48], [49]. The pattern recovered from the mean estimates of node times indicates a nested model with an overall down-shift associated with the most basal moths followed by a series of up-shifts corresponding to the major clades Glossata (moths with a proboscis) and Ditrysia (moths with partitioned female reproductive tracts). The pattern of shifts within the advanced moths and butterflies is poorly resolved with a number of events showing limited robustness with respect to branch length variation. If shift recovery across multiple samples of the Markov Chain Monte Carlo used in dating is considered ([24], see Supplementary materials), several of these events are found to be collapsed into a single shift associated with the redefined Obtectomera [48], [49] (Fig. S3, Table S4), which also corresponds to the shift associated with second largest improvement in overall model likelihood in single-shift models (Table 1, see Supplementary materials for further discussion). Comparable previous work on patterns of diversification within Diptera identified a series of nested shifts within the order that are not recovered in our study [14]. These differences can be attributed to the placement of radiations within a more inclusive phylogenetic context, i.e. within Holometabola in its entirety, resulting in greater estimated turnover within the group, as well as minor differences in taxonomic sampling and dating between analyses. Contrary to previous views, which have tended to emphasize the role of particular ecologies (notably phytophagy) [11], [10] in determining patterns of hexapod richness, we find no evidence that the upshifted groups are associated with a particular set of life history traits. Instead, our results suggest diverse responses within the Mesozoic insect fauna to the ecological transition and novel opportunities provided by the Cretaceous angiosperm expansion [2].

## Discussion

Ultimate explanations of insect diversification can be classified into morphological key innovations and ecological interactions [3]. Our results highlight the importance of complete metamorphosis as the major key innovation underpinning the pattern of hexapod species richness. The mechanism by which complete metamorphosis promotes diversification is incompletely understood. However, previous workers have suggested that the ecological division of adult and juvenile life stages separated by a pupal stage in Holometabola may play a major role [3], [4], [9]. The adaptation to novel ecological niches likely played a role in promoting diversity within specific hexapod radiations, such as family-level or lower taxonomic levels, but there is no evidence here to support the idea that a single suite of ecological traits is generally associated with shifts in hexapod diversification. Instead, the patterns observed are consistent with distinct members of the community responding in a wide variety of ways to the ecological changes following the angiosperm radiation and continuing to the present day. However, we did find evidence that the radiation of angiosperms itself triggered a number of upshifts in diversification rate across both non-holometabolan and holometabolan groups, marking the evolution of angiosperms as a key ecological change in the evolutionary history of Hexapoda. It is important to note that our recognition of these patterns is dependent on the inferred phylogenetic topology, which contains some regions of considerable phylogenetic uncertainty (see Supplementary materials). However, it is unlikely that the major findings of our analysis – i.e. key roles of complete metamorphosis and angiosperm evolution as well as the failure to recover a distinct suite of ecological traits underlying a species group's phylogenetic richness – will change in the light of future improvements to the topology, dating, and extant species richness of the insect phylogenetic tree, which collectively will combine to further improve our understanding of the origins and diversification of this key component of terrestrial ecosystems.

## Materials and Methods

The phylogeny of 874 terminal taxa, representing familial groups within Hexapoda was inferred based on eight widely sampled molecular markers including nuclear (CAD, Ef1α, PGD) and mitochondrial (COI, COII) protein coding sequences and 16S, 18S and 28S rRNA sequences. All included taxa are chimeric, i.e. the sequences used are assembled from multiple individuals and species, such as to reduce the problem of non-overlapping sampling within the source datasets and to maximize gene coverage for each of the sampled terminals. All gene partitions were aligned using MAFFT [50] with the exception of 18S and 28S rRNA, which were aligned using an automated profile alignment based on the structural reference database SILVA [51]. Conserved regions identified using the Gblocks protocol [52] and third codon positions for all the protein coding genes were excluded due to saturation. In total the concatenated sequence had a length of 7021bp and was 50.69% complete at the nucleotide level. Topological relationships within hexapods were inferred using a constrained maximum likelihood analysis in RAxML [22], [23] implemented on the CIPRES web cluster [53]. Data was partitioned by nucleotide position and genome for protein coding sequences with the three ribosomal partitions each modeled independently. The tree topology was constrained in order to ensure comparability with other recent phylogenetic studies and to control the behavior of under-sampled and unstable taxa. The implemented constraints and further details relating to phylogenetic inference are described in Supplementary materials.

The fully resolved maximum likelihood topology estimated above was used as the basis for a relaxed independent gamma rates clock implemented in Mr. Bayes 3.2 [24]. Calibration was based on 86 fossils listed in Table S1, and implemented as hard minimum bounds on the ages of the relevant nodes with a hard maximum bound taken from a recent comparable molecular clock study [26]. Chains were run for 12 million generations with sampling conducted every 500 generations and a burn-in fraction of 50%. Further details are listed in Supplementary materials.

Estimates of extant richness for terminal taxa were compiled from recent encyclopedic sources (Supplementary materials). Models of clade diversification were implemented in R v 2.15.1 [54]. Topological shifts in diversification rate were identified using the stepwise algorithm MEDUSA [36] (package TurboMEDUSA [55]) on the dated consensus tree. Likelihood ratio tests to compare optimal placement of the first rate shift were implemented in the package Laser [56] using turnover estimates taken from MEDUSA. Estimates of richness of clades in the absence of rate shifts were calculated in the package Geiger [57], . The consistency of inferred shifts with respect to uncertainties in the node ages was accessed across 500 randomly sampled trees taken from the post-burnin phase of the dating chains. Further discussion of diversification analyses can be found in the Supplementary materials.

## Supporting Information

Figure S1 **Nodal support on the phylogeny.** Nodes marked with circles are either constrained (red) or have high bootstrap support (light blue 50–80%, dark blue: over 80%).
(TIF)

Figure S2 **Topology showing 95% confidence intervals on node ages (transparent blue bars).** Black rings denote 100 Ma intervals from the present. Nodes denoted with red circles are involved in calibration (see Table S2 for details).
(TIF)

Figure S3 **The fifty shifts with the highest rates of recovery in samples from the MCMC chain (Table S4) plotted together on the tree topology.** Shifts are denoted as Fig. 1 with novel shifts not recovered on the mean tree denoted by red circles. Groupings on the ring and other information are as Fig.1.
(TIF)

Table S1 **Estimates of extant species richness for terminal groups and GenBank accession numbers for sequences used in phylogenetic reconstruction.**
(XLSX)

Table S2 **Fossil calibrations implemented in dating the tree topology.** Calibrated nodes are plotted on Fig. S2. Where available, radiometric date estimates are referenced on the first occurrence of the deposit. Alternatively, the relevant stage termination is given based on ([1]-Supplementary references). References to cited studies in Supplementary materials.
(XLSX)

Table S3 **Parameter values and shifts in species richness associated with MEDUSA model shifts inferred across the mean topology.** Shifts listed here are plotted on Fig. 1. Richness shifts are plotted on Fig. 3. See text and Supplementary materials for further discussion.
(XLSX)

Table S4 **The fifty most robustly recovered shifts inferred from 500 samples from the post-burnin Markov Chain Monte Carlo (MCMC).** Shifts are plotted on Figure S3. Shifts without equivalents on the mean tree are highlighted in bold.
(XLSX)

Datafile S1 **Tree topology in Nexus format.** Contains two tree files with a) an undated cladogram including bootstrap support for nodes and b) a dated topology including confidence intervals for the node ages and denotation of inferred shift events matching Fig. 1.
(TXT)

Datafile S2 **Alignment and MrBayes instructions in Nexus format.** Contains the implemented alignment after processing with the commands and priors used in setting the MrBayes dating run.
(TXT)

Text S1 **Supplementary Experimental Procedures and Discussion.** Contains further details of experimental procedures, discussion of topology and reliability of diversification shift estimates, and cited references for fossil calibrations and species richness estimates (Table S1, S2).
(DOCX)
