# Supplementary figures and images for: Phylogenetic Distribution of Extant Richness Suggests Metamorphosis Is a Key Innovation Driving Diversification in Insects
Source: PLoS One. 2014 Oct 2;9(10):e109085. doi: 10.1371/journal.pone.0109085 (PMC4183542; doi:10.1371/journal.pone.0109085)

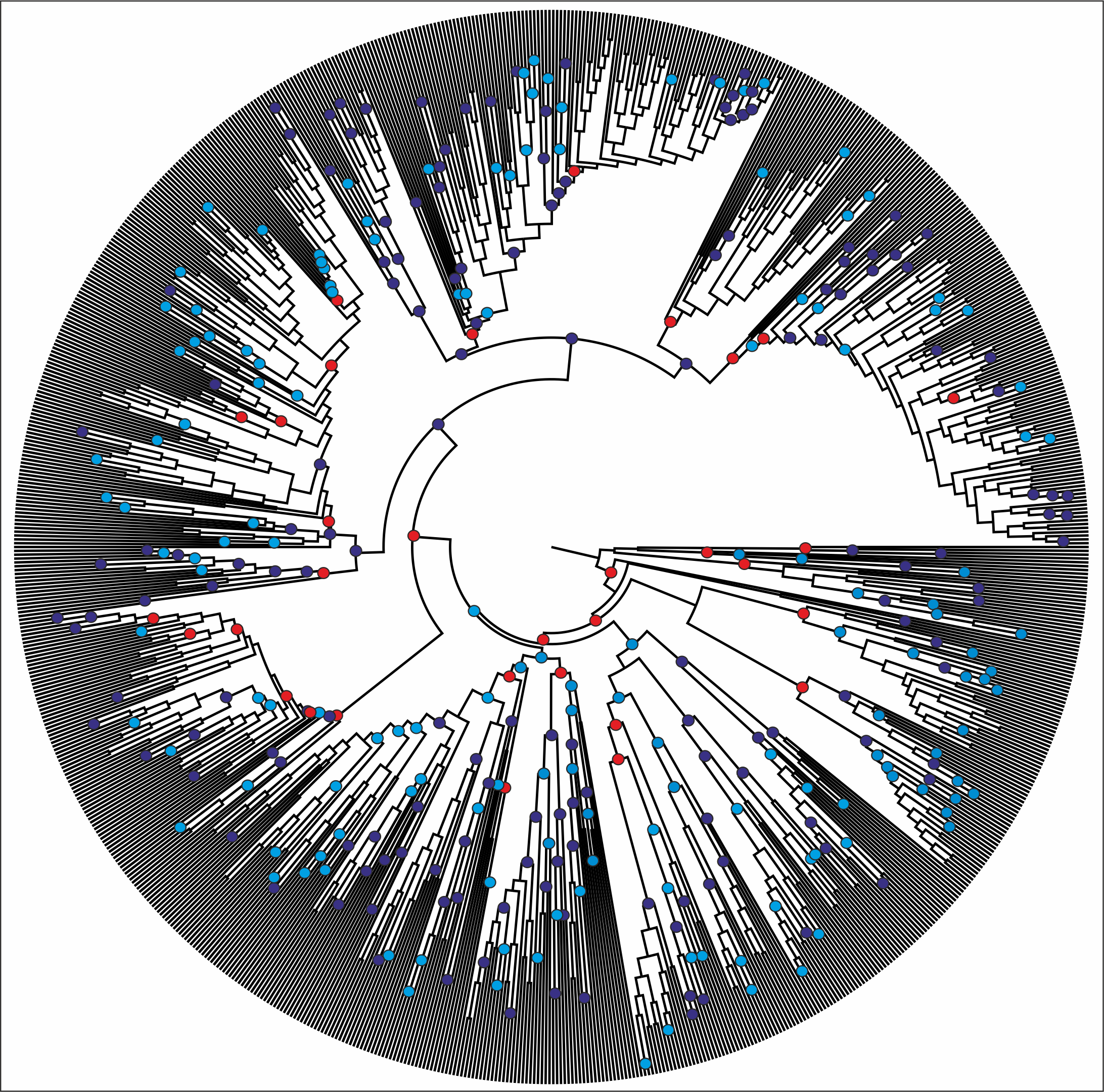

Supplement: Figure S1 — Nodal support on the phylogeny. Nodes marked with circles are either constrained (red) or have high bootstrap support (light blue 50–80%, dark blue: over 80%). (TIF) [file pone.0109085.s001.tif]

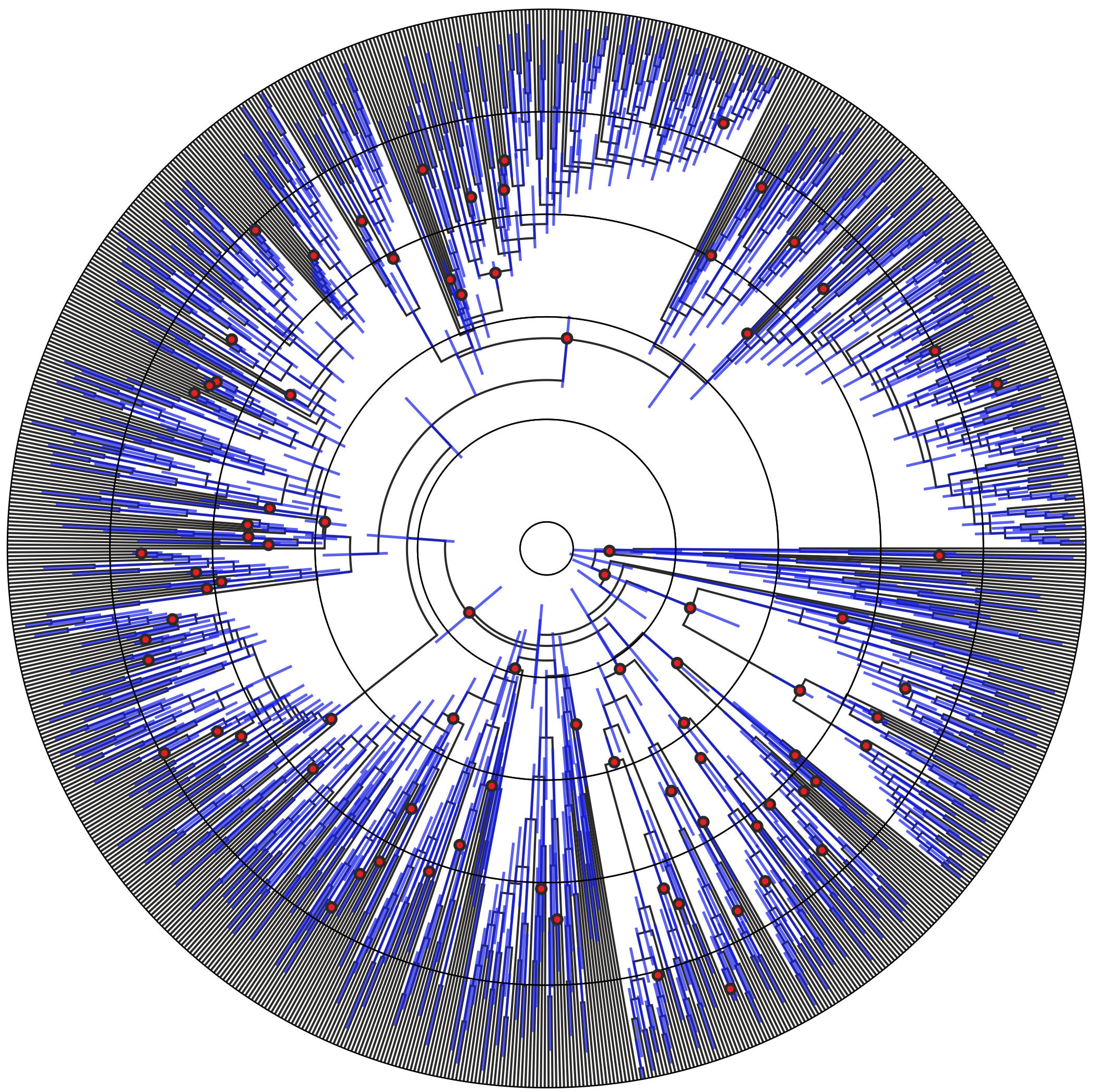

Supplement: Figure S2 — Topology showing 95% confidence intervals on node ages (transparent blue bars). Black rings denote 100 Ma intervals from the present. Nodes denoted with red circles are involved in calibration (see Table S2 for details). (TIF) [file pone.0109085.s002.tif]

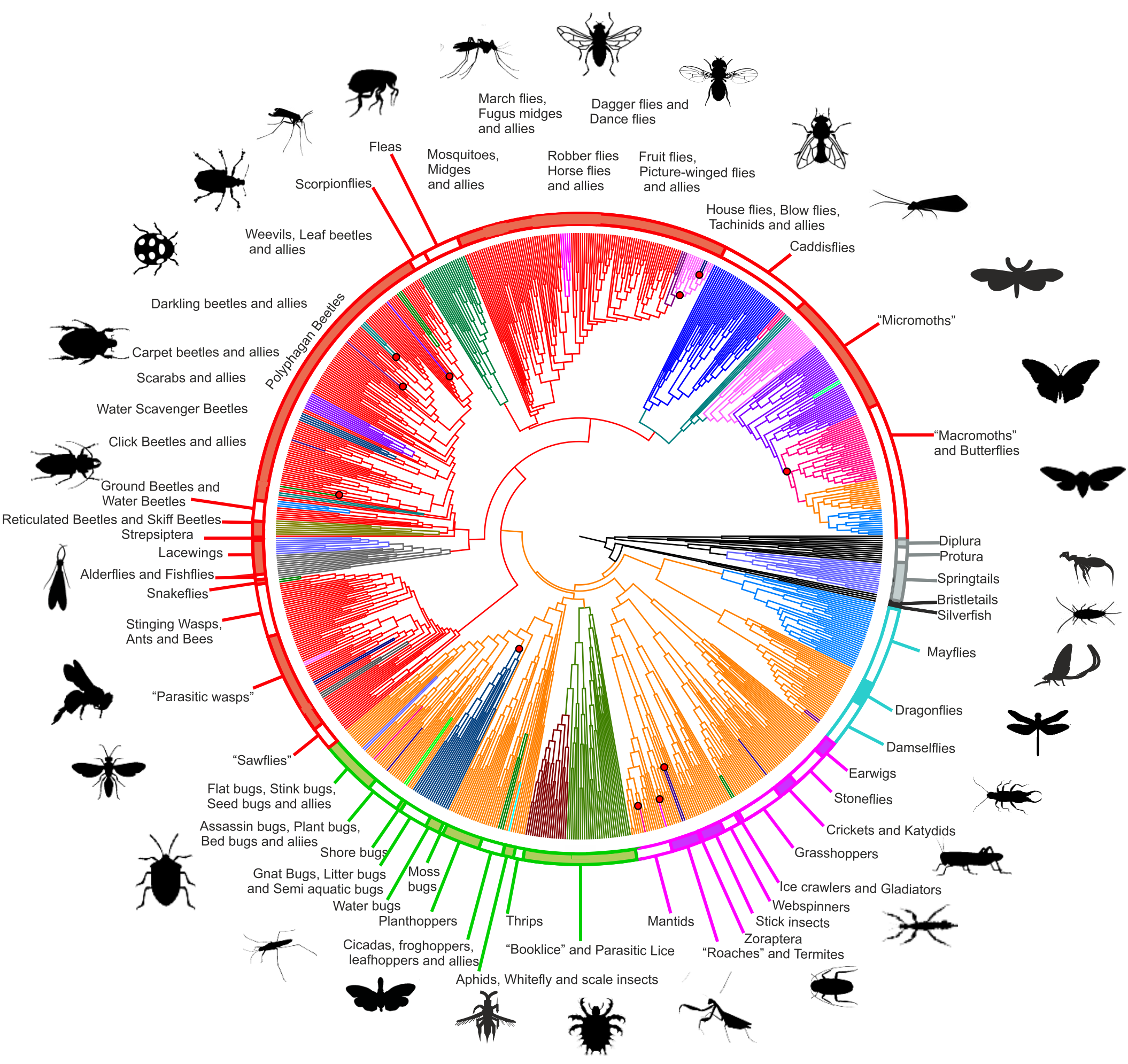

Supplement: Figure S3 — The fifty shifts with the highest rates of recovery in samples from the MCMC chain (Table S4) plotted together on the tree topology. Shifts are denoted as Fig. 1 with novel shifts not recovered on the mean tree denoted by red circles. Groupings on the ring and other information are as Fig.1. (TIF) [file pone.0109085.s003.tif]
