# Supplementary material for: Phylogenetic Distribution of Extant Richness Suggests Metamorphosis Is a Key Innovation Driving Diversification in Insects
Source: PLoS One. 2014 Oct 2;9(10):e109085. doi: 10.1371/journal.pone.0109085 (PMC4183542; doi:10.1371/journal.pone.0109085)
Supplement: Text S1 — Supplementary Experimental Procedures and Discussion. Contains further details of experimental procedures, discussion of topology and reliability of diversification shift estimates, and cited references for fossil calibrations and species richness estimates (Table S1, S2). (DOCX) [file pone.0109085.s010.docx]

## Supplementary Experimental Procedures and Discussion

## Phylogenetic reconstruction

In recent years the application of molecular techniques has revolutionized our understanding of a number of key issues in insect phylogenetics, notably with respect to Holometabola [74]. However there remain considerable regions of uncertainty regarding relationships within the group [105],[106]. Given that our aim was to explore patterns of diversification in a global phylogenetic context this necessitated the construction of a novel dated phylogenetic framework based on available sequence data.

As noted in the main text we collected sequence data for 874 terminal taxa. Data was extracted from GenBank, and the curated ribosomal database SILVA [107] and extracted using BLAST [108] searches targeted on *Drosophila* sequences of the listed genes, taken from previous phylogenetic studies and a published complete mitochondrial genome (ref AJ400907) [109]. Sequence data from multiple studies and individuals were combined in order to maximize coverage across the different studied markers (i.e. the terminal taxa used are chimeras), with sampling favoring those with highest similarity to the BLAST target (Accession numbers given in Table S1, Full alignment after processing in Datafile S2). This approach enables maximum coverage across lineages on available markers and so minimizes missing data and improves phylogenetic accuracy [110],[111]. For Hexapoda such a procedure was seen as especially beneficial, as the choice of sequenced markers varies widely across studies, and previous work has indicated the importance of combining multiple data sources for accurate phylogenetic reconstruction within the group, which contains a number of areas of significant phylogenetic uncertainty [105],[106],[112]. The justification for chimera construction is contingent on the assertion of monophyly for the terminal lineages involved [111]. Systematic testing of the monophyletic status of the hexapod families and super-families included here is beyond the scope of this study, and requires high resolution phylogenies of the various lineages involved (e.g. [113]-[115]), however it is the authors opinion that in using the most up to date hexapod taxonomy available, we reflect current hypotheses regarding relations within the group, and so provide a benchmark against which future improvements in taxonomic understanding can be examined.

Most hexapod groups were resolved to the family level, consistent with the resolution of available data for the fossil record and estimates of extant species richness. In order to maintain consistency across genetic partitions taxonomy followed that of Genbank up to August 2013. Exceptions to the family level resolution include the Hemiptera suborder Sternorrhyncha (Aphidoidea (10 families), Coccoidea (23 families), Phylloxeroidea (2 families) and Psylloidea (7 families)) and the small parasitic order Strepsiptera (7 families). In the former case this was the result of taxonomic conflict between Genbank and the consulted sources of species richness estimates, while in the case of the latter this represented a deliberate strategy to minimize problems of long branch attraction known to be associated with this taxon [116]-[118].

An overview of the treatment of data is provided in the main text. The eight markers sampled (CAD, Ef1α, PGD, COI, COII and 16S, 18S and 28S rRNA) were individually aligned using MAFFT (local pair distances, max iterations=1000) [119], with the exceptions of 18S and 28S which were aligned using an automated profile alignment based on the structural reference database SILVA [107]. This approach was selected due to the combination of time efficiency and the preservation of conserved structural elements that previous studies have shown to be significant in accurate phylogenetic reconstruction within Hexapoda [120],[121]. All partitions were subjected to Gblocks [122] in order to remove regions of poor alignment with the minimum conserved block length set to three for protein coding genes and two for ribosomal sequences. Third codon positions for all protein-coding sequences were excluded due to the risk of substitution saturation [123]. In total the concatenated sequence had a length of 7021bp and was 50.69% complete at the nucleotide level.

Missing data presents a potentially serious issue for accurate inferences of phylogenetic relationships due to the potential for missing sites to contribute to long-branch attraction, which pulls together unrelated terminals within the topology [124]. Recent simulations and empirical studies have, however, shown that the impact of such issues is strongly dependent on the structure of the missing information [124],[125], and that sparsely sampled matrices can still generate accurate phylogenetic estimates for large clades [126]-[128]. The impact of missing data on molecular clock estimates is less clearly understood and appears to be variable with respect to different nodes within a topology with the overall effect being strongly dependent on the structure of the missing information [129]. There are also possible interactions with sequence saturation, a process that is also known to generate noise in clock date estimates [130],[131]. Due to the nature of the available data i.e. incongruence in the availability of markers within Genbank across different hexapod groups, the direct impact of missing data on the findings presented here is difficult to assess. However, recent work has suggested that relaxed Bayesian procedures, such as the one implemented in our analysis, are relatively insensitive to issues of data composition compared with other dating protocols [129],[132].

Due to the large number of taxa involved, simultaneous inference of topology and divergence times (e.g. using BEAST [133] or MrBayes [134] ) was not computationally feasible. As a result we adopted a two phase analysis whereby the rapid maximum likelihood routine RAxML [135],[136] implemented on the CIPRES webserver [137], was used to infer topology within the constraints described below, and the resulting tree was subsequently dated using MrBayes 3.2 [134]. The dated topology and an undated cladogram with bootstrap support values are provided in Datafile S1. For RAxML, data was partitioned such that codons one and two for nuclear and mitochondrial genes respectively were allotted separate GTR+CAT models, as were each of the three RNA partitions included. Reported bootstrap values are calculated using an MR-based stopping criterion [136] resulting in 650 replicated bootstrap samples.

## Constraints on Topology

Recent studies attempting to infer broad scale phylogenies for diverse groups, have typically relied on a tiered system of phylogenetic inference, such that datasets with restricted sampling have been used to provide a backbone in order to guide the placement of further taxa e.g. [127],[138]. For Hexapoda the distribution of sequence information currently available in GenBank means that there are restrictions on the availability of data for the most controversial nodes within the tree and as a result a backbone approach fails to adequately control the placement of unstable taxa (see [105],[112] for review). We instead adopted a constraint-based approach whereby relationships that have been recovered as consistently well-supported relationships documented in other studies were used to guide the reconstruction of the tree used here. In order to define suitable constraints the available phylogenetic literature for hexapods was reviewed since 2005, and used to define suitable constraints in accordance with the following principals:

- A constraint must reflect a recognized systematic group that is widely accepted within the literature of the appropriate taxa, defined by reference to appropriate encyclopedic sources e.g. [26],[139].
- Recovery with strong support, defined as bootstrap support of at least 95 under maximum likelihood or parsimony, or alternatively Bayesian posterior probability of 0.99, in all relevant recent molecular phylogenic studies with sufficient resolution to inform the family level analysis conducted here. Where both maximum likelihood and Bayesian trees were available the former were favored due to the well known tendency for Bayesian analyses to over-inflate confidence values [140] and in order to maximize methodological comparability with the process of topological inference used here.

Constraints meeting these criteria are listed below with example references that include the most recent and comprehensive taxonomic treatments of the relevant groups. In addition to these we also constrained the monophyly of recognized orders with the following exceptions where paraphyly is known or suspected [105],[106]: “Blattodea” w.r.t Isoptera [141], “Pscoptera” w.r.t Pthiraptera [142] and “Mecoptera” with respect to. Siphonaptera [143] and possibly Phasmatodea w.r.t. Embiodea [144],[145].

Finally, we also used constraints to restrict the movement of representatives of the unstable polyneopteran order Zoraptera which was extremely data deficient with respect to the studied markers and whose position was strongly influenced by biases induced by the uniquely modified nuclear rRNAs of this taxon [146],[147],[121]. Traditional classifications placed Zoraptera at the base of Paraneoptera (e.g. [148]), however recent opinion favors an unresolved placement somewhere within Polyneoptera [120],[146],[147],[149]-[153]. The constraint used here was based on the most recent study, and the only one to be based on protein-coding markers independent of the problematic rRNAs, which recovered Zoraptera as sister to Dictyoptera [153], a position which is also accepted in a recent review of hexapod ordinal relationships [105]. This position was accepted over other recent proposals including a sister group relationship with Embiodea (“Mystroptera”) [150],[151] or Dermaptera (“Haplocercata”) [149] due to lack of molecular support for the former, and to issues surrounding data quality in the single study reporting the later [147]. In the absence of constraint Zoraptera was recovered as sister to all other Neoptera, a position that has never been supported in previous work and which has serious consequences for the pattern of diversification due to low richness of this lineage.

**Implemented constraints (see also Fig. S1)**

- **Ordinal relationships**- reviewed in [105],[106]: Insecta, Dicondylia, Pterygota, Neoptera, Holometabola
- **Hymenoptera** [115],[154],[155]: Unicalcarida, Vespina, Aculeata, Chrysidoidea, Anthophila
- **Coleoptera** [113],[156]: Hydrophiloidea, Scarabaeoidea, Cucujiformia, Curculionoidea
- **Diptera** [114],[157],[158]: Brachycera, Schizophora
- **Lepidoptera** [159]-[161]: Glossata, Rhopalocera

## Dating the tree

Dating of the topology generated in RAxML was conducted using an independent gamma rates clock implemented in MrBayes 3.2 [134]. The data were partitioned as above and optimal models for each partition identified using PHYML, implemented in the TOPALI browser [162]. The form of clock implemented was based on Bayes factor comparisons conducted on preliminary data including incomplete versions of the data matrix [163]. Priors on the clock rates were based on those used in a comparable multi-gene analysis of Hymenoptera [134] (clock rate lognormal mean= 6.10, SD 2.458582, independent gamma rates variation exponential mean= 37.12). Preliminary runs of the Bayesian chain identified a strong tendency for chains to become stuck on distinct local optima resulting in poor convergence and inadequate parameter sampling. In order to resolve this, runs were conducted under low temperature conditions (heating parameter 0.005) with the proposal frequencies of a number of parameters being modified in order to achieve acceptance rates within the recommended range (20-80%) (Datafile S2) [164]. Markov Chain Monte Carlo (MCMC) chains were run for 12 million generations with sampling conducted every 500 generations with a burn-in fraction of 50% necessary to remove the impact of all the suboptimal peaks obtained during sampling. Convergence of the runs was assessed based on trace outputs analyzed in Tracer v1.5 [133],[165] and on the adequate sampling of the majority of the model parameters (average effective sample sizes >200 and potential scale reduction factors of approximately 1, implying that the independent runs generated approximately identical parameter values suggesting that they converge on the underlying distribution)[134],[164]. Dating was performed locally on a Mac 3.4Ghz Intel Core i7 processor and required approximately 1100 hours to run to completion.

Calibration was conducted using 86 fossils taken from the recent palaeoentomological literature (Table S2). In defining calibrations we favored specific named fossils following the recommendations of [166]. All calibrations were implemented as hard minimum bounds on a uniform distribution with a hard maximum based on the upper 95% CI on the basal divergence in Hexapoda in [167]. The same maximum was also placed on the age of the root node. The appropriate implementation of fossil calibrations for molecular clock studies remains an active area of debate [168] and we recognize that previous work has shown that the use of uniform calibrations can lead to non-uniform effective priors [169]. However we feel that, due to the incomplete nature of the hexapod fossil record [170], attempts to explicitly calibrate node age maxima using probability distributions of fossil absences (e.g. [171]) introduce unknowable levels of error, and consequently favor uniform distributions as making minimal assumptions regarding maximum node age. Note that this is one of a number of different approaches to calibration that have been previously implemented with respect to dating divergence in Hexapoda and that all of these have produced broadly comparable results with regard to the age of major events [167],[171]-[173]. Where available minimum radiometric dates for the relevant deposits have been used, otherwise deposits were treated as the age of the termination of the relevant stage in [1] (Table S2).

## Diversification analyses

All analyses of diversification and tree processing were conducted in R v 2.15.1 [174]. Estimates of extant species richness for terminal taxa were sourced from previous publications [113],[175]-[179], recent encyclopedias [26],[139],[180],[181] and online taxonomic resources [182]-[192] (Table S1)*.* Where taxonomic sources conflicted with the classification given here species assigned to any subgroups were deducted from the more inclusive clade.

The primary algorithm used to infer the topological position of shifts in diversification rate was the stepwise greedy ML algorithm MEDUSA [193] as implemented in the package TurboMEDUSA [194]. This algorithm proceeds by estimating optimal parameters for a global birth death model on a given tree with tip richnesses and then going through all nodes and identifying the optimal position of a break in the diversification model (henceforth a “shift”) that maximally improved the overall AICc score. This process is repeated adding further shifts until some threshold is achieved where there is no further improvement in AICc. The appropriate threshold score was calculated internally to the routine and given as an improvement in AICc of 9.320989 units. The resulting optimal model identified 48 shift events with parameter values listed in Table S3.

In order to estimate the impact of particular shift events on the overall richness of hexapods we used simulated birth death models to estimate what the richness of clades would have been had the modeled shifts not occurred. This was done using the crown.limits [195] function in package Geiger [196] with parameter values taken from the parental model estimated in MEDUSA (i.e. the model within which the focal shift is nested) and node age based on the consensus tree. The function gives the upper and lower confidence intervals on the richness of a clade of that age, from which a mean estimate was calculated. This was compared with both the raw estimates of clade richness (Fig. S4) and estimates of richness which had been corrected for the presence of further shifts in diversification rate, by replacing the richness of nodes with subject to further shifts with their mean modeled richness (Fig. 4).

The reported shift on Holometabola is of particular interest as it is both the first shift recovered by the greedy MEDUSA algorithm, implying that its inclusion makes the greatest overall improvement to AICc scores and it is also significant in ideas relating to key innovations in driving hexapod diversification [197]. In order to explore the importance of this shift and to make comparisons with other potential key innovations we examined the likelihood improvement associated with a range of shifts using the package laser [198]. The functions fitNDR_1rate and fitNDR_2rate were used to find parameter estimates and log likelihood values for the optimal global model and models with a single shift at every possible node respectively. In both cases these functions require that the turnover parameter be specified and this was based on the value obtained from for the homogenous model (i.e. that with no shifts) estimated in MEDUSA (eps= 0.9990712). Parameter estimates and likelihood scores of the resulting models are presented in Table 1, with an emphasis placed on groups associated with potential shifts in diversification rate identified in previous studies [199]-[201]. The results of likelihood ratio tests are shown for comparison of the Holometabola shift model with these alternatives and the single shift model.

In order to access the degree to which uncertainty with respect to node age impacted on the pattern of rate shift events the analysis was repeated across 500 random samples taken from the post-burin MCMC chain used in dating. Samples were taken so as to be evenly distributed between the two parallel chains and were scaled into time units using the appropriate estimates of the overall clock rate. Note that due to the use of the two-stage phylogenetic inference process described above it was not possible to access the impact of topological uncertainty on the results of this study. The occurrence of a shift associated with a particular node across the different samples was scored and the proportion of samples in which a node occurs is used as the basis for the coloration of the symbols on Fig. 1 and is reported in Table S3. Table S4 lists the top 50 nodes with respect to proportional occurrence and these are depicted on Fig. S3. For this study we have elected not to use the alterative diversification model TreePar [138],[202],[203] due to limitations on the available computational resources which restricted the capacity to simulate the large numbers of species complete trees needed to calculate appropriate confidence intervals [202].

## Tree Topology

The tree used in this study combines data in the form of widely sampled molecular markers with information from previous studies in the form of imposed constraints (see above and Fig. S1). The result is a topology that while broadly consistent with current view in entomology remains only weakly supported in certain regions. In many ways this is a reflection on the current state of molecular entomology, a field in which significant progress has been made in terms of some well studied relationships [105],[106] but that as yet has not led to overall stability, particularly with respect to regions reflecting rapid ancient divergence within the group [112]. The aim here was to summarize the available information into a coherent form that could be used for macro-evolutionary inference, however we recognize that further targeted studies are required to resolve outstanding issues within poorly resolved regions of the tree.

At an ordinal level the present topology is broadly consistent with the current consensus regarding hexapod relationships [105],[106]. Regions of strong support include increasingly well established relationships within Holometabola, with Hymenoptera as basal to two super-ordinal groupings termed Neuropteroidea (Neuropterida, Coleoptera, Strepsiptera) and Mecopterida, with the later including two traditional groupings, Amphiesmenoptera (Trichoptera and Lepidoptera) and Antliophora (Diptera, “Mecoptera” and Siphonaptera) reviewed in [74],[105],[204], all of which are recovered with strong (over 80% bootstrap support (BS)). With respect to controversial regions among the basal winged insects we recover extremely weak support (BS 20) for a monophyletic Palaeoptera [112] consistent with recent morphological [205],[206] and genomic findings [207], as well as modest support for the monophyly of Polyneoptera (BS 59) and Paraneoptera (BS 70) (the later disputed in [153]). Within Polyneoptera few conclusions can be drawn regarding ordinal relationships (partially due to the constraint imposed on Zoraptera- see above) although we do find strong evidence for an unrecognized grouping of Plecoptera and Dermaptera (BS 96) that is also present on [120],[121],[153],[208], Xenonomia (Grylloblatodea + Mantophasmatodea , BS 93) [149], as well as weak support for Eukinolabia (Phasmatodea +Embioptera BS 58) [149],[145]. In terms of possible paraphyletic orders our tree supported the accepted relationships [105],[106] of Siphonaptera within Mecoptera (Boreidae sister to Siphonaptera (BS 80)) [143], Pthiraptera within Pscoptera (BS 54, the former weakly recovered as monophyletic (BS 68) [142],[209]), Isoptera within Blattodea (BS 71) [141],[210],[211],[212]. Due to the constraints implemented we did not test the potential relationship of Strepsiptera within Coleoptera with the later being constrained as monophyletic [117],[213],[214].

Within orders comparison with previous work becomes more difficult as many groups have received relatively little study making the impact of difference in the use of markers and methodology difficult to assess. Here we restrict comment to relationships within the four megadiverse holometabolan (Hymenoptera, Coleoptera, Diptera and Lepidoptera) orders as these have received the most study of any hexapod groups and have the greatest consequence for understanding diversification. Available trees for all four groups include a mix of regions of consistently high support (often incorporated here as constraints- see above) and regions where available markers fail to resolve divergences. Examples of the latter include basal relationships among Apocrita (Hym.), particularly with respect to members of the former superfamiles “Proctotrupoidea” [115],[215] and “Vespoidea” [154],[216]), the “backbone” relationships of Polyphaga (Col.) [113] and the affinities of many taxa in Schizophora (Dip.) (notably within Cyclorrhapha) [114],[157] and Apoditrysia (Lep.) [128],[160],[161] Unsurprisingly this study, which draws on sequences generated in the above work, demonstrates comparable regions of uncertainty, although occasionally with minor modifications reflecting differences in taxonomic sampling and methodology. In many ways this reflects the limits of current hexapod phylogenetics although the use of constraints to fix well supported nodes ensure that at least these, many of which are in some way associated within an inferred shift, are comparable across the different studies. Despite recent progress it seems likely that it will be some years and requiring targeted sequencing before these issues are completely addressed, at which point the impact of this improved resolution on the inferred pattern of diversification can be accessed.

## Perspectives on diversification from sampling the MCMC

The majority of studies involved in modeling diversification rates within dated trees have tended to focus exclusively on patterns associated with the mean tree without any consideration of the potential uncertainties involved in estimating node ages. Bayesian methods as implemented here provide a natural way to approximate the confidence intervals on the node ages through the use of samples from the postdated MCMC (see methods). Comparing the pattern of nodes consistently recovered from such samples (the top fifty of which are shown on Fig. S3 and listed in Table S4) provides an alternative insight into the processes of diversification active within the group. Overall the pattern of well supported shifts is broadly consistent with that recovered on the mean tree indicating that most of the inferred events, including the shifts associated with Holometabola and Pterygota, are relatively robust with respect to uncertainties in branch length. However there are also a number of differences within certain major clades that change our perspective on diversification patterns.

The majority of changes in the pattern of diversification occur within the mega-diverse Holometabolan orders. In Lepidoptera the pattern of shifts alters such that in place of the idiosyncratic shifts associated with butterflies (Rhopalocera) and Gelechioidea a more secure shift is recovered associated with the redefined Obtectomera, which encompasses both these groups as well as macromoths, and is one of the best supported clades in the advanced Lepidoptera [128],[160],[161]. This shift also corresponds to that identified as the second best position under the two-rate model in Table 1. Within Coleoptera four novel shifts are highlighted involving three large and recently derived phytophagous groups [113],[217]: Buprestidae (jewel beetles) , Curculionidae (“true” weevils) and Mordellidae (tumbling flower beetles) (the last in association with the also tending to phytophagous Anthicidae (ant-like flower beetles) and the parasitoid Meloidae (Blister beetles)) [113],[218], as well as the large detritivorous family Tenebrionidae (Darkling beetles). Within Diptera the key role of Calyptratae, and Tachinidae in particular, in dominating the pattern of diversification is again emphasized. Tachinids are among the most diverse (9626 described species) [180] and youngest (divergence estimated as 26.27 Ma, CIs 9.96-42.31 Ma) fly families, implying exceptional rates of diversification, which may reflect the group’s successful adaptation to a parasitoid lifestyle on a huge variety of arthropod hosts, particularly similarly recently derived Lepidoptera [219].

Outside of Holometabola there are also minor modifications to the apparent pattern of diversification including a potential shift associated with the aquatic associated bugs (including Nepomorpha, Gerromorpha, Dipsocoromorpha and Enicocephalomorpha), modifications to the apparent pattern within Dictyoptera that highlight three super rich clades (i.e. Blattidae + Blaberidae (Blattodea), Mantidae (Mantodea), Termitidae (Isoptera)) and bring results more in line with those of previous studies [220], and the loss of idiosyncratic up-shifts associated with a sub-clade of Ephemeroptera and Neanuridae (Collembola). As well as highlighting further candidates for radiations within Hexapoda these results also emphasize the potential dangers of relying on a single set of date estimates when discussing diversification, as the resulting pattern may include shifts shaped by idiosyncrasies of the particular tree chosen and so not be representative of the overall pattern implied by the data.

**Supplementary References (Continuous from Table S2)**

1. Gradstein FM, Ogg G, Schmitz M (2012) The Geologic Time Scale 2012. Elsevier. 1176 p.

2. Parry SF, Noble SR, Crowley QG, Wellman CH (2011) A high-precision U–Pb age constraint on the Rhynie Chert Konservat-Lagerstatte: time scale and other implications. J Geol Soc Lond 168: 863–872.

3. Whalley P, Jarzembowski EA (1981) A new assessment of Rhyniella, the earliest known insect, from the Devonian of Rhynie, Scotland. Nature 291: 317–317. doi:10.1038/291317a0.

4. Greenslade P, Whalley PES (1996) The systematic position of Rhyniella Praecursor Hirst and Maulik (Collembola). The earliest known Hexapod. 2nd International Seminar on Apterygota. University of Siena.

5. Wilson HM, Martill DM (2001) A new Japygid Dipluran from the Lower Cretaceous of Brazil. Palaeontology 44: 1025–1031. doi:10.1111/1475-4983.00213.

6. Engel MS, Grimaldi DA (2004) New light shed on the oldest insect. Nature 427: 627–630. doi:10.1038/nature02291.

7. Riek E, Kukalova-Peck J (1984) A new interpretation of dragonfly wing venation based upon Early Upper Carboniferous fossils from Argentina (Insecta: Odonatoidea) and basic character states in pterygote wings. Can J Zool 62: 1150–1166.

8. Spalletti LA, Fanning CM, Rapela C. (2008) Dating the Triassic continental rift in the southern Andes: the Potrerillos Formation, Cuyo Basin, Argentina. Geol Acta 6: 267–283.

9. Davis RB, Nicholson DB, Saunders EL, Mayhew PJ (2011) Fossil gaps inferred from phylogenies alter the apparent nature of diversification in dragonflies and their relatives. BMC Evol Biol 11: 252. doi:10.1186/1471-2148-11-252.

10. Carpenter FM (1960) A Triassic Odonate From Argentina. Psyche J Entomol 67: 71–75. doi:10.1155/1960/23289.

11. Liu Y, Liu Y, Ji S, Yang Z (2006) U-Pb zircon age for the Daohugou Biota at Ningcheng of Inner Mongolia and comments on related issues. Chin Sci Bull 51: 2634–2644. doi:10.1007/s11434-006-2165-2.

12. Huang D-Y, Nel A (2009) First Chinese Cymatophlebiidae from the Middle Jurassic of Inner Mongolia (Odonata: Anisoptera: Aeshnoptera). Palaeodiversity 2: 199–204.

13. Vasilenko DV (2005) New Damselflies (Odonata: Synlestidae, Hemiphlebiidae) from the Mesozoic Transbaikalian Locality of Chernovskie Kopi. Paleontol J 39: 280.

14. McCafferty WP (1997) Discovery and analysis of the oldest mayflies (Insecta, Ephemeroptera) known from amber. Bull Soc Hist Nat Toulouse 133: 77–82.

15. Martill DM, Bechly G, Loveridge RF (2007) The Crato fossil beds of Brazil: Window into an ancient world. Cambridge University Press. 572 p.

16. Prokop J, Nel A, Hoch I (2005) Discovery of the oldest known Pterygota in the Lower Carboniferous of the Upper Silesian Basin in the Czech Republic (Insecta: Archaeorthoptera). Geobios 38: 383–387. doi:10.1016/j.geobios.2003.11.006.

17. Béthoux O, Nel A (2002) Venation pattern and revision of Orthoptera sensu nov. and sister groups. Phylogeny of Palaeozoic and Mesozoic Orthoptera sensu nov. Zootaxa 96: 1–88.

18. Béthoux O, Nel A, Lapeyrie J, Gand G, Galtier J (2002) Raphogla rubra gen. n. sp. n. the oldest representative of the clade of modern Ensifera (Orthoptera: Tettigoniidea, Gryllidea). Eur J Entomol 99: 111–116.

19. Gorochov AV, Jarzembowski EA, Coram RA (2006) Grasshoppers and crickets (Insecta: Orthoptera) from the Lower Cretaceous of southern England. Cretac Res 27: 641–662. doi:10.1016/j.cretres.2006.03.007.

20. Gorochov AV (2005) Review of Triassic Orthoptera with descriptions of new and little known taxa: Part 2. Paleontol J 39: 272–279.

21. Heads SW (2008) The First Fossil Proscopiidae (Insecta Orthoptera Eumastacoidea) with comments on the historical biogeography and evolution of the family. Palaeontology 51: 499–507. doi:10.1111/j.1475-4983.2008.00756.x.

22. Béthoux O, Wieland F (2009) Evidence for Carboniferous origin of the order Mantodea (Insecta: Dictyoptera) gained from forewing morphology. Zool J Linn Soc 156: 79–113. doi:10.1111/j.1096-3642.2008.00485.x.

23. Vršanský P (2005) Lower Cretaceous cockroaches and mantids (Insecta: Blattaria, Mantodea) from the Sharin-Gol in Mongolia. Entomol Probl 35: 163–167.

24. Engel MS, Grimaldi DA, Krishna K (2007) Primitive termites from the Early Cretaceous of Asia (Isoptera). Stuttg Beitr Zur Naturkunde Ser B Geol Paläontol 371: 1–32.

25. Engel MS, Grimaldi DA, Krishna K (2009) Termites (Isoptera): Their phylogeny, classification, and rise to ecological dominance. Am Mus Novit 3650: 1–27.

26. Grimaldi DA, Engel MS (2005) Evolution of the Insects. Cambridge University Press.

27. Nel A, Roy R (1996) Revision of fossil “mantid” and “ephemerid” species described by Piton from the Paleocene of Menat (France) Mantodea Chaeteessidae; Ensifera Tettigonioidea. Eur J Entomol 93: 223–234.

28. Grimaldi DA (2003) A revision of Cretaceous mantises and their relationships, including new taxa (Insecta, Dictyoptera, Mantodea). Am Mus Novit 3412: 1–47.

29. Huang D-Y, Nel A (2009) Oldest webspinners from the Middle Jurassic of Inner Mongolia, China (Insecta: Embiodea). Zool J Linn Soc 156: 889–895. doi:10.1111/j.1096-3642.2008.00499.x.

30. Shi G, Grimaldi DA, Harlow GE, Wang J, Wang J, et al. (2012) Age constraint on Burmese amber based on U–Pb dating of zircons. Cretac Res 37: 155–163. doi:10.1016/j.cretres.2012.03.014.

31. Engel MS, Grimaldi DA (2006) The earliest webspinners (Insecta: Embiodea). Am Mus Novit 3514: 1–15. doi:10.1206/0003-0082(2006)3514[1:TEWIE]2.0.CO;2.

32. Wedmann S, Bradler S, Rust J (2007) The first fossil leaf insect: 47 million years of specialized cryptic morphology and behavior. Proc Natl Acad Sci 104: 565–569. doi:10.1073/pnas.0606937104.

33. Huang D, Nel A, Zompro O, Waller A (2008) Mantophasmatodea now in the Jurassic. Naturwissenschaften 95: 947–952. doi:10.1007/s00114-008-0412-x.

34. Béthoux O, Cui Y, Kondratieff B, Stark B, Ren D (2011) At last, a Pennsylvanian stem-stonefly (Plecoptera) discovered. BMC Evol Biol 11: 248. doi:10.1186/1471-2148-11-248.

35. Liu Y, Sinitshenkova ND, Ren D (2009) A revision of the Jurassic stonefly Genera Dobbertiniopteryx Ansorge and Karanemoura Sinitshenkova (Insecta: Plecoptera), with the description of new species from the Daohugou locality, China. Paleontol J 43: 183–190. doi:10.1134/S0031030109020099.

36. Swisher CC, Wang Y, Wang X, Xu X, Wang Y (1999) Cretaceous age for the feathered dinosaurs of Liaoning, China. Nature 400: 58–61. doi:10.1038/21872.

37. Sun G, Dilcher DL, Wang H, Chen Z (2011) A eudicot from the Early Cretaceous of China. Nature 471: 625–628. doi:10.1038/nature09811.

38. Yushuang L, Dong R, Sinitshenkova ND, Chungkun S (2008) Three New Stoneflies (Insecta: Plecoptera) from the Yixian Formation of Liaoning, China. Acta Geol Sin - Engl Ed 82: 249–256. doi:10.1111/j.1755-6724.2008.tb00575.x.

39. Engel MS (2011) New earwigs in mid-Cretaceous amber from Myanmar (Dermaptera, Neodermaptera). ZooKeys: 137–152. doi:10.3897/zookeys.130.1293.

40. Nel P, Azar D, Prokop J, Roques P, Hodebert G, et al. (2012) From Carboniferous to Recent: wing venation enlightens evolution of thysanopteran lineage. J Syst Palaeontol 10: 385–399. doi:10.1080/14772019.2011.598578.

41. Grimaldi DA, Engel MS (2006) Fossil Liposcelididae and the lice ages (Insecta: Psocodea). Proc R Soc B Biol Sci 273: 625–633. doi:10.1098/rspb.2005.3337.

42. Yoshizawa K, Lienhard C (2010) In search of the sister group of the true lice: A systematic review of booklice and their relatives, with an updated checklist of Liposcelididae (Insecta: Psocodea). Arthropd Syst Phylogeny 68: 181–195.

43. Grimaldi DA, Engel MS (2006) Extralimital fossils of the “Gondwanan” family Sphaeropsocidae (Insecta, Psocodea). Am Mus Novit 3523: 1–18.

44. Nel A, Waller A (2007) The first fossil Compsocidae from Cretaceous Burmese amber (Insecta, Psocoptera, Troctomorpha). Cretac Res 28: 1039–1041. doi:10.1016/j.cretres.2007.02.002.

45. Fraser N, Grimaldi DA, Olsen PE, Axsmith B (1996) A Triassic Lagerstatte from eastern North America. Nature 380: 615–619.

46. Yao Y, Cai W, Ren D (2007) Pristinochterus gen. n.(Hemiptera: Ochteridae) from the Upper Mesozoic of northeastern China. Eur J Entomol 104: 827.

47. Yao Y, Zhang W, Ren D, Shih C (2011) New fossil Ochteridae (Hemiptera: Heteroptera: Ochteroidea) from the Upper Mesozoic of north-eastern China, with phylogeny of the family. Syst Entomol 36: 589–600.

48. Azar D, Nel A (2010) The earliest fossil schizopterid bug (Insecta: Heteroptera) in the Lower Cretaceous amber of Lebanon. Ann Société Entomol Fr 46: 193–197.

49. Zhang W, Yao Y, Ren D (2011) New shore bug (Hemiptera, Heteroptera, Saldidae) from the Early Cretaceous of China with phylogenetic analyses. ZooKeys: 185–198. doi:10.3897/zookeys.130.1563.

50. Perrichot V, Nel A, Neraudeau D (2005) Gerromorphan bugs in Early Cretaceous French amber (Insecta: Heteroptera): first representatives of Gerridae and their phylogenetic and palaeoecological implications. Cretac Res 26: 793–800. doi:10.1016/j.cretres.2005.05.003.

51. Lin Q (1992) Late Triassic insect fauna from Toksun Xinjiang. Acta Palaeont Sin 31: 313–335.

52. Yao Y, Ren D, Rider DA, Cai W (2012) Phylogeny of the infraorder Pentatomomorpha based on fossil and extant morphology, with description of a new fossil family from China. PLoS ONE 7: e37289. doi:10.1371/journal.pone.0037289.

53. Scherbakov DE (2009) Review of fossil and extant genera of the cicada family Tettigarctidae. Russ Entomol J 17: 343–348.

54. Szwedo J (2007) Glisachaemus jonasdamzeni gen. et sp. nov. of Cixiidae from the Eocene Baltic amber (Hemiptera: Fulgoromorpha). Alavasia 1: 109–116.

55. Szwedo J, Wang B, Zhang H (2011) An extraordinary Early Jurassic planthopper from Hunan (China) representing a new family Qiyangiricaniidae fam. nov. (Hemiptera: Fulgoromorpha: Fulgoroidea). Acta Geol Sin - Engl Ed 85: 739–748. doi:10.1111/j.1755-6724.2011.00479.x.

56. Szwedo J, Nel A (2011) The oldest aphid insect from the Middle Triassic of the Vosges, France. Acta Palaeontol Pol 56: 757–766.

57. Nel A, Roques P, Nel P, Prokop J, Steyer JS (2007) The earliest holometabolous insect from the Carboniferous: a “crucial” innovation with delayed success (Insecta Protomeropina Protomeropidae). Ann Société Entomol Fr 43: 349–355.

58. Labandeira CC (2011) Evidence for an Earliest Late Carboniferous Divergence Time and the Early Larval Ecology and Diversification of Major Holometabola Lineages. Entomol Am 117: 9–21. doi:10.1664/10-RA-011.1.

59. Ronquist F, Klopfstein S, Vilhelmsen L, Schulmeister S, Murray DL, et al. (2012) A total-evidence approach to dating with fossils, applied to the early radiation of the Hymenoptera. Syst Biol 61: 973–999. doi:10.1093/sysbio/sys058.

60. Rasnitsyn AP, Zhang H (2004) Composition and age of the Daohugou hymenopteran (Insecta, Hymenoptera - Vespida) assemblage from Inner Mongolia, China. Palaeontology 47: 1507–1517.

61. Shih C, Feng H, Liu C, Zhao Y, Ren D (2010) Morphology, phylogeny, evolution, and dispersal of pelecinid wasps (Hymenoptera: Pelecinidae) Over 165 million years. Ann Entomol Soc Am 103: 875–885. doi:10.1603/AN09043.

62. Wang X, Zhou Z, He H, Jin F, Wang Y, et al. (2005) Stratigraphy and age of the Daohugou Bed in Ningcheng, Inner Mongolia. Chin Sci Bull 50: 2369–2376. doi:10.1007/BF03183749.

63. Shih C, Feng H, Ren D (2011) New fossil Heloridae and Mesoserphidae Wasps (Insecta, Hymenoptera, Proctotrupoidea) from the Middle Jurassic of China. Ann Entomol Soc Am 104: 1334–1348. doi:10.1603/AN10194.

64. Azevedo CO, Azar D (2012) A new fossil subfamily of Bethylidae (Hymenoptera) from the Early Cretaceous Lebanese amber and its phylogenetic position. Zool Curitiba 29: 210–218. doi:10.1590/S1984-46702012000300004.

65. Liu Z, Engel MS, Grimaldi DA (2007) Phylogeny and geological history of the cynipoid wasps (Hymenoptera: Cynipoidea). Am Mus Novit 3583: 1–48.

66. Engel MS, Grimaldi DA (2006) The first Cretaceous Spider Wasp (Hymenoptera: Pompilidae). J Kans Entomol Soc 79: 359–368.

67. Poinar Jr G (2009) Mellittosphex (Hymenoptera:Melittosphecidae), a Primitive Bee not a a Wasp. Palaeontology 52: 483–484. doi:10.1111/j.1475-4983.2008.00840.x.

68. Wichard W, Engel MS (2006) A new alderfly in Baltic amber (Megaloptera, Sialidae). Am Mus Novit 3513: 1–9.

69. Wedmann S, Makarkin VN (2007) A new genus of Mantispidae (Insecta: Neuroptera) from the Eocene of Germany, with a review of the fossil record and palaeobiogeography of the family. Zool J Linn Soc 149: 701–716. doi:10.1111/j.1096-3642.2007.00273.x.

70. Yang Q, Makarkin VN, Ren D (2010) Remarkable New Genus of Gumillinae (Neuroptera: Osmylidae) From the Jurassic of China. Ann Entomol Soc Am 103: 855–859.

71. Ren D, Engel MS (2008) A second antlion from the Mesozoic of northeastern China (Neuroptera: Myrmeleontidae). Alavasia 2: 183–186.

72. Makarkin VN, Yang Q, Peng Y, Ren D (2012) A comparative overview of the neuropteran assemblage of the Lower Cretaceous Yixian Formation (China), with description of a new genus of Psychopsidae (Insecta: Neuroptera). Cretac Res 35: 57–68.

73. Kukalová-Peck J, Beutel RG (2012) Is the Carboniferous Adiphlebia lacoana really the “oldest beetle”? Critical reassessment and description of a new Permian beetle family. Eur J Entomol 109: 633–645.

74. Beutel RG, Friedrich F, Hörnschemeyer T, Pohl H, Hünefeld F, et al. (2011) Morphological and molecular evidence converge upon a robust phylogeny of the megadiverse Holometabola. Cladistics 27: 341–355. doi:10.1111/j.1096-0031.2010.00338.x.

75. Martins-Neto RG, Gallego O, Mancuso A (2006) The Triassic insect fauna from Argentina. Coleoptera from the Los Rastros Formation (Bermejo Basin), La Rioja Province. Ameghiniana 43: 591–609.

76. Cai C, Short AEZ, Huang D (2012) The first Skiff Beetle (Coleoptera: Myxophaga: Hydroscaphidae) from Early Cretaceous Jehol Biota. J Paleontol 86: 116–119.

77. Chatzimanolis S, Grimaldi DA, Engel MS, Fraser N (2012) Leehermania prorova, the earliest staphyliniform beetle, from the Late Triassic of Virginia (Coleoptera: Staphylinidae). Am Mus Novit 3761: 1–28.

78. Bai M, Ren D, Yang X (2012) Prosinodendron krelli from the Yixian Formation, China: A missing link among Lucanidae, Diphyllostomatidae and Passalidae (Coleoptera: Scarabaeoidea). Cretac Res 34: 334–339. doi:10.1016/j.cretres.2011.11.017.

79. Nikolajev GV, Wang B, Liu Y, Zhang H (2011) Stag Beetles from the Mesozoic of Inner Mongolia, China (Scrabaeoidea: Lucanidae). Acta Palaeont Sin 50: 41–47.

80. Nikolajev GV, Ren D (2010) The oldest fossil Ochodaeidae (Coleoptera: Scarabaeoidea) from the Middle Jurassic of China. Zootaxa 2553: 65–68.

81. Yu Y, Leschen RAB, Slipinski A, Ren D, Pang H (2012) The first fossil Bark-Gnawing Beetle from the Middle Jurassic of Inner Mongolia, China (Coleoptera: Trogossitidae). Ann Zool 62: 245–252. doi:10.3161/000345412X652765.

82. Fikáček M, Prokin A, Angus RB, Ponomarenko A, Yue Y, et al. (2012) Revision of Mesozoic fossils of the helophorid lineage of the superfamily Hydrophiloidea (Coleoptera: Polyphaga). Acta Entomol Musei Natl Pragae 52: 89–127.

83. Fikáček M, Prokin A, Angus RB, Ponomarenko A, Yue Y, et al. (2012) Phylogeny and the fossil record of the Helophoridae reveal Jurassic origin of extant hydrophiloid lineages (Coleoptera: Polyphaga). Syst Entomol 37: 420–447. doi:10.1111/j.1365-3113.2012.00630.x.

84. Gratshev V, Legalov A (2011) New mesozoic Ithyceridae beetles (Coleoptera). Paleontol J 45: 77–82. doi:10.1134/S0031030111010060.

85. Poinar Jr G, Kirejtshuk AG, Buckley R (2008) Pleuroceratos burmiticus, n. gen., n. sp.(Coleoptera: Silvanidae) from early Cretaceous Burmese amber. Proc Entomol Soc Wash 110: 250–257.

86. Kirejtshuk AG, Azar D (2008) New taxa of beetles (Insecta, Coleoptera) from Lebanese amber with evolutionary and systematic comments. Alavesia 2: 15–46.

87. Aristov D, Bashkuev A (2008) New insects (Insecta: Mecoptera, Grylloblattida) from the Middle Permian Chepanikha locality, Udmurtia. Paleontol J 42: 159–165. doi:10.1007/s11492-008-2006-7.

88. Minet J, Huang D-Y, Nel A (2010) Early Mecopterida and the systematic position of the Microptysmatidae (Insecta: Endopterygota). Ann Société Entomol Fr 46: 262–270.

89. Ansorge J (2003) Upper Liassic Amphiesmenopterans (Trichoptera + Lepidoptera) from Germany – a review. Acta Zool Cracoviensia 46: 285–290.

90. Hao W, Diying H (2012) A New Species of Liadotaulius (Insecta: Trichoptera) from the Middle Jurassic of Daohugou, Inner Mongolia. Acta Geol Sin - Engl Ed 86: 320–324. doi:10.1111/j.1755-6724.2012.00662.x.

91. Sukatsheva ID, Jarzembowski EA (2001) Fossil caddisflies (Insecta: Trichoptera) from the Early Cretaceous of southern England II. Cretac Res 22: 685–694. doi:10.1006/cres.2001.0292.

92. Wichard W, Ross E, Ross AJ (2011) Palerasnitsynus gen. n. (Trichoptera, Psychomyiidae) from Burmese amber. ZooKeys: 323–330. doi:10.3897/zookeys.130.1449.

93. Sohn J-C, Labandeira CC, Davis DR, Mitter C (2012) An annotated catalog of fossil and subfossil Lepidoptera (Insecta: Holometabola) of the world. Zootaxa 3286: 1–132.

94. Labandeira C, Dilcher DL, Davis DR, Wagner DL (1994) Ninety-seven million years of angiosperm-insect association: Paleobiological insights into the meaning of coevolution. Proc Natl Acad Sci U S A 91: 12278–12282.

95. Huang D, Engel MS, Cai C, Wu H, Nel A (2012) Diverse transitional giant fleas from the Mesozoic era of China. Nature 483: 210–204. doi:10.1038/nature10839.

96. Gao T, Shih C, Xu X, Wang S, Ren D (2012) Mid-Mesozoic flea-like ectoparasites of feathered or haired vertebrates. Curr Biol 22: 732–735. doi:10.1016/j.cub.2012.03.012.

97. Yang X-G, Shih C-K, Ren D, Petrulevičius JF (2012) New Middle Jurassic hangingflies (Insecta: Mecoptera) from Inner Mongolia, China. Alcheringa Australas J Palaeontol 36: 195–201. doi:10.1080/03115518.2012.622143.

98. Blagoderov V, Grimaldi DA, Fraser N (2007) How time flies for flies: Diverse Diptera from the Triassic of Virginia and early radiation of the order. Am Mus Novit 3572: 1–39.

99. Lukashevich ED, Przhiboro AA, Marchal-Paper F, Grauvogel-Stamm L (2010) The oldest occurrence of immature Diptera (Insecta), Middle Triassic, France. Ann Société Entomol Fr 46: 4–22.

100. Lukashevich ED, Huang D-Y, Lin Q-B (2006) Rare families of lower Diptera (Hennigmatidae, Blephariceridae, Perissommatidae) from the Jurassic of China. Stud Dipterol 13: 127–143.

101. Krzemiñski W, Krzemiñski E (2003) Triassic Diptera: descriptions, revisions and phylogenetic relations. Acta Zool Cracoviensia 46: 153–184.

102. Zhang J, Li H (2012) New taxa of snipe flies (Diptera: Brachycera: Rhagionidae) in the Daohugou Biota, China. Paleontol J 46: 157–163. doi:10.1134/S0031030112020128.

103. Zhang J (2012) New horseflies and water snipe-flies (Diptera: Tabanidae and Athericidae) from the Lower Cretaceous of China. Cretac Res 36: 1–5. doi:10.1016/j.cretres.2012.01.004.

104. Winkler IS, Labandeira C, Wappler T, Wilf P (2010) Distinguishing Agromyzidae (Diptera) leaf mines in the fossil record: New taxa from the Paleogene of North America and Germany and their evolutionary implications. J Paleontol 84: 935–954.

105. Trautwein MD, Wiegmann BM, Beutel RG, Kjer KM, Yeates DK (2012) Advances in insect phylogeny at the dawn of the postgenomic era. Annu Rev Entomol 57: 449–468.

106. Yeates DK, Cameron SL, Trautwein M (2012) A view from the edge of the forest: recent progress in understanding the relationships of the insect orders. Aust J Entomol 51: 79–87. doi:10.1111/j.1440-6055.2012.00857.x.

107. Pruesse E, Quast C, Knittel K, Fuchs BM, Ludwig W, et al. (2007) SILVA: a comprehensive online resource for quality checked and aligned ribosomal RNA sequence data compatible with ARB. Nucleic Acids Res 35: 7188 –7196. doi:10.1093/nar/gkm864.

108. Altschul SF, Gish W, Miller W, Myers EW, Lipman DJ (1990) Basic local alignment search tool. J Mol Biol 215: 403–410. doi:10.1016/S0022-2836(05)80360-2.

109. Azou Y, Bregliano J-C (2001) I–R system of hybrid dysgenesis in Drosophila melanogaster: analysis of the mitochondrial DNA in reactive strains exhibiting different potentials for I factor transposition. Heredity 86: 110–116. doi:10.1046/j.1365-2540.2001.00814.x.

110. Springer MS, Scally M, Madsen O, de Jong WW, Douady CJ, et al. (2004) The use of composite taxa in supermatrices. Mol Phylogenet Evol 30: 883–884. doi:10.1016/j.ympev.2003.09.019.

111. Campbell V, Lapointe F-J (2009) The Use and Validity of Composite Taxa in Phylogenetic Analysis. Syst Biol 58: 560–572. doi:10.1093/sysbio/syp056.

112. Whitfield JB, Kjer KM (2008) Ancient Rapid Radiations of Insects: Challenges for Phylogenetic Analysis. Annu Rev Entomol 53: 449–472. doi:10.1146/annurev.ento.53.103106.093304.

113. Hunt T, Bergsten J, Levkanicova Z, Papadopoulou A, John OS, et al. (2007) A comprehensive phylogeny of beetles reveals the evolutionary origins of a superradiation. Science 318: 1913 –1916. doi:10.1126/science.1146954.

114. Wiegmann BM, Trautwein MD, Winkler IS, Barr NB, Kim J-W, et al. (2011) Episodic radiations in the fly tree of life. Proc Natl Acad Sci 108: 5690–5695. doi:10.1073/pnas.1012675108.

115. Heraty J, Ronquist F, Carpenter JM, Hawks D, Schulmeister S, et al. (2011) Evolution of the hymenopteran megaradiation. Mol Phylogenet Evol 60: 73–88. doi:10.1016/j.ympev.2011.04.003.

116. Huelsenbeck JP (1998) Systematic bias in phylogenetic analysis: Is the Strepsiptera problem solved? Syst Biol 47: 519–537.

117. Niehuis O, Hartig G, Grath S, Pohl H, Lehmann J, et al. (2012) Genomic and Morphological Evidence Converge to Resolve the Enigma of Strepsiptera. Curr Biol 22: 1309–1313. doi:10.1016/j.cub.2012.05.018.

118. Pohl H, Beutel RG (2005) The phylogeny of Strepsiptera (Hexapoda). Cladistics 21: 328–374. doi:10.1111/j.1096-0031.2005.00074.x.

119. Katoh K, Misawa K, Kuma K, Miyata T (2002) MAFFT: a novel method for rapid multiple sequence alignment based on fast Fourier transform. Nucleic Acids Res 30: 3059 –3066. doi:10.1093/nar/gkf436.

120. Misof B, Niehuis O, Bischoff I, Rickert A, Erpenbeck D, et al. (2007) Towards an 18S phylogeny of hexapods: Accounting for group-specific character covariance in optimized mixed nucleotide/doublet models. Zoology 110: 409–429. doi:10.1016/j.zool.2007.08.003.

121. Wang Y, Engel MS, Rafael JA, Dang K, Wu H, et al. (2013) A Unique Box in 28S rRNA Is Shared by the Enigmatic Insect Order Zoraptera and Dictyoptera. PLoS ONE 8: e53679. doi:10.1371/journal.pone.0053679.

122. Talavera G, Castresana J (2007) Improvement of phylogenies after removing divergent and ambiguously aligned blocks from protein sequence alignments. Syst Biol 56: 564 –577. doi:10.1080/10635150701472164.

123. Ho SYW, Jermiin LS (2004) Tracing the Decay of the Historical Signal in Biological Sequence Data. Syst Biol 53: 623–637. doi:10.1080/10635150490503035.

124. Sanderson MJ, McMahon MM, Steel M (2010) Phylogenomics with incomplete taxon coverage: the limits to inference. BMC Evol Biol 10: 155. doi:10.1186/1471-2148-10-155.

125. Wiens JJ (2003) Missing data, incomplete taxa, and phylogenetic accuracy. Syst Biol 52: 528 –538. doi:10.1080/10635150390218330.

126. Burleigh JG, Hilu KW, Soltis DE (2009) Inferring phylogenies with incomplete data sets: a 5-gene, 567-taxon analysis of angiosperms. BMC Evol Biol 9: 61. doi:10.1186/1471-2148-9-61.

127. Wiens JJ, Fetzner JW, Parkinson CL, Reeder TW (2005) Hylid frog phylogeny and sampling strategies for speciose clades. Syst Biol 54: 778–807. doi:10.1080/10635150500234625.

128. Cho S, Zwick A, Regier JC, Mitter C, Cummings MP, et al. (2011) Can Deliberately Incomplete Gene Sample Augmentation Improve a Phylogeny Estimate for the Advanced Moths and Butterflies (Hexapoda: Lepidoptera)? Syst Biol 60: 782–796. doi:10.1093/sysbio/syr079.

129. Douzery EJP, Snell EA, Bapteste E, Delsuc F, Philippe H (2004) The timing of eukaryotic evolution: Does a relaxed molecular clock reconcile proteins and fossils? Proc Natl Acad Sci U S A 101: 15386–15391. doi:10.1073/pnas.0403984101.

130. Soubrier J, Steel M, Lee MSY, Sarkissian CD, Guindon S, et al. (2012) The Influence of Rate Heterogeneity among Sites on the Time Dependence of Molecular Rates. Mol Biol Evol 29: 3345–3358. doi:10.1093/molbev/mss140.

131. Zheng Y, Peng R, Kuro-o M, Zeng X (2011) Exploring Patterns and Extent of Bias in Estimating Divergence Time from Mitochondrial DNA Sequence Data in a Particular Lineage: A Case Study of Salamanders (Order Caudata). Mol Biol Evol 28: 2521–2535. doi:10.1093/molbev/msr072.

132. Mulcahy DG, Noonan BP, Moss T, Townsend TM, Reeder TW, et al. (2012) Estimating divergence dates and evaluating dating methods using phylogenomic and mitochondrial data in squamate reptiles. Mol Phylogenet Evol 65: 974–991. doi:10.1016/j.ympev.2012.08.018.

133. Drummond A, Rambaut A (2007) BEAST: Bayesian evolutionary analysis by sampling trees. BMC Evol Biol 7: 214.

134. Ronquist F, Teslenko M, van der Mark P, Ayres DL, Darling A, et al. (2012) MrBayes 3.2: Efficient Bayesian phylogenetic inference and model choice across a large model space. Syst Biol 61: 539–542. doi:10.1093/sysbio/sys029.

135. Stamatakis A, Ludwig T, Meier H (2005) RAxML-III: A fast program for maximum likelihood-based inference of large phylogenetic trees. Bioinformatics 21: 456–463. doi:10.1093/bioinformatics/bti191.

136. Stamatakis A, Hoover P, Rougemont J (2008) A rapid bootstrap algorithm for the RAxML web servers. Syst Biol 57: 758–771.

137. Miller MA, Pfeiffer W, Schwartz T (2010) Creating the CIPRES Science Gateway for inference of large phylogenetic trees. Proceedings of the Gateway Computing Environments Workshop. New Orleans: GCE. pp. 1–8.

138. Jetz W, Thomas GH, Joy JB, Hartmann K, Mooers AO (2012) The global diversity of birds in space and time. Nature 491: 444–448. doi:10.1038/nature11631.

139. Resh VH, Cardé RT (2009) Encyclopedia of Insects, Second Edition. Elsevier.

140. Alfaro ME, Zoller S, Lutzoni F (2003) Bayes or Bootstrap? A Simulation Study Comparing the Performance of Bayesian Markov Chain Monte Carlo Sampling and Bootstrapping in Assessing Phylogenetic Confidence. Mol Biol Evol 20: 255 –266. doi:10.1093/molbev/msg028.

141. Inward D, Beccaloni G, Eggleton P (2007) Death of an order: a comprehensive molecular phylogenetic study confirms that termites are eusocial cockroaches. Biol Lett 3: 331 –335. doi:10.1098/rsbl.2007.0102.

142. Yoshizawa K, Johnson KP (2010) How stable is the “Polyphyly of Lice” hypothesis (Insecta: Psocodea)?: A comparison of phylogenetic signal in multiple genes. Mol Phylogenet Evol 55: 939–951. doi:10.1016/j.ympev.2010.02.026.

143. Whiting MF (2002) Mecoptera is paraphyletic: multiple genes and phylogeny of Mecoptera and Siphonaptera. Zool Scr 31: 93–104. doi:10.1046/j.0300-3256.2001.00095.x.

144. Zrzay J (2008) Four chapters about the monophyly of insect “orders”: A review of recent phylogenetic contributions. Acta Entomol Musei Natl Pragae 48: 217–232.

145. Friedemann K, Wipfler B, Bradler S, Beutel RG (2012) On the head morphology of Phyllium and the phylogenetic relationships of Phasmatodea (Insecta). Acta Zool 93: 184–199. doi:10.1111/j.1463-6395.2010.00497.x.

146. Yoshizawa K, Johnson KP (2005) Aligned 18S for Zoraptera (Insecta): Phylogenetic position and molecular evolution. Mol Phylogenet Evol 37: 572–580. doi:10.1016/j.ympev.2005.05.008.

147. Yoshizawa K (2010) Direct optimization overly optimizes data. Syst Entomol 35: 199–206. doi:10.1111/j.1365-3113.2010.00526.x.

148. Hennig W (1969) Stammesgeschichte der Insekten. Kramer, Frankfurt am Main.

149. Terry MD, Whiting MF (2005) Mantophasmatodea and phylogeny of the lower neopterous insects. Cladistics 21: 240–257. doi:10.1111/j.1096-0031.2005.00062.x.

150. Friedrich F, Beutel RG (2008) The thorax of Zorotypus (Hexapoda, Zoraptera) and a new nomenclature for the musculature of Neoptera. Arthropod Struct Dev 37: 29–54. doi:10.1016/j.asd.2007.04.003.

151. Yoshizawa K (2011) Monophyletic Polyneoptera recovered by wing base structure. Syst Entomol 36: 377–394. doi:10.1111/j.1365-3113.2011.00572.x.

152. Simon S, Narechania A, DeSalle R, Hadrys H (2012) Insect Phylogenomics: Exploring the Source of Incongruence Using New Transcriptomic Data. Genome Biol Evol 4: 1295–1309. doi:10.1093/gbe/evs104.

153. Ishiwata K, Sasaki G, Ogawa J, Miyata T, Su Z-H (2011) Phylogenetic relationships among insect orders based on three nuclear protein-coding gene sequences. Mol Phylogenet Evol 58: 169–180. doi:10.1016/j.ympev.2010.11.001.

154. Debevec AH, Cardinal S, Danforth BN (2012) Identifying the sister group to the bees: a molecular phylogeny of Aculeata with an emphasis on the superfamily Apoidea. Zool Scr 41: 527–535. doi:10.1111/j.1463-6409.2012.00549.x.

155. Wilson JS, Dohlen CD von, Forister ML, Pitts JP (2013) Family-Level Divergences in the Stinging Wasps (Hymenoptera: Aculeata), with Correlations to Angiosperm Diversification. Evol Biol 40: 101–107. doi:10.1007/s11692-012-9189-0.

156. Lawrence JF, Ślipiński A, Seago AE, Thayer MK, Newton AF, et al. (2011) Phylogeny of the Coleoptera Based on Morphological Characters of Adults and Larvae. Ann Zool 61: 1–217. doi:10.3161/000345411X576725.

157. Caravas J, Friedrich M (2013) Shaking the Diptera tree of life: performance analysis of nuclear and mitochondrial sequence data partitions. Syst Entomol 38: 93–103. doi:10.1111/j.1365-3113.2012.00657.x.

158. Lambkin CL, Sinclair BJ, Pape T, Courtney GW, Skevington JH, et al. (2013) The phylogenetic relationships among infraorders and superfamilies of Diptera based on morphological evidence. Syst Entomol 38: 164–179. doi:10.1111/j.1365-3113.2012.00652.x.

159. Regier JC, Zwick A, Cummings MP, Kawahara AY, Cho S, et al. (2009) Toward reconstructing the evolution of advanced moths and butterflies (Lepidoptera: Ditrysia): an initial molecular study. BMC Evol Biol 9: 280. doi:10.1186/1471-2148-9-280.

160. Mutanen M, Wahlberg N, Kaila L (2010) Comprehensive Gene and Taxon Coverage Elucidates Radiation Patterns in Moths and Butterflies. Proc R Soc B Biol Sci 277: 2839–2848. doi:10.1098/rspb.2010.0392.

161. Regier JC, Mitter C, Zwick A, Bazinet AL, Cummings MP, et al. (2013) A Large-Scale, Higher-Level, Molecular Phylogenetic Study of the Insect Order Lepidoptera (Moths and Butterflies). PLoS ONE 8: e58568. doi:10.1371/journal.pone.0058568.

162. Milne I, Lindner D, Bayer M, Husmeier D, McGuire G, et al. (2009) TOPALi v2: a rich graphical interface for evolutionary analyses of multiple alignments on HPC clusters and multi-core desktops. Bioinformatics 25: 126–127. doi:10.1093/bioinformatics/btn575.

163. Xie W, Lewis PO, Fan Y, Kuo L, Chen M-H (2011) Improving marginal likelihood estimation for Bayesian phylogenetic model selection. Syst Biol 60: 150 –160. doi:10.1093/sysbio/syq085.

164. Ronquist F, van der Mark P, Huelsenbeck JP (2009) Bayesian phylogenetic analysis using MrBayes. The Phylogenetic Handbook: a Practical Approach to Phylogenetic Analysis and Hypothesis Testing. Cambridge University Press.

165. Rambaut A, Drummond AJ (2009) Tracer [computer program]. Available: http://tree.bio.ed.ac.uk/software/tracer/.

166. Parham JF, Donoghue PCJ, Bell CJ, Calway TD, Head JJ, et al. (2012) Best practices for justifying fossil calibrations. Syst Biol 61: 346–359. doi:10.1093/sysbio/syr107.

167. Rota-Stabelli O, Daley AC, Pisani D (2013) Molecular timetrees reveal a Cambrian colonization of land and a new scenario for Ecdysozoan evolution. Curr Biol 23: 392–398. doi:10.1016/j.cub.2013.01.026.

168. Ho SYW, Phillips MJ (2009) Accounting for calibration uncertainty in phylogenetic estimation of evolutionary divergence times. Syst Biol 58: 367 –380. doi:10.1093/sysbio/syp035.

169. Heled J, Drummond AJ (2011) Calibrated tree priors for relaxed phylogenetics and divergence time estimation. Syst Biol 61: 138–149. doi:10.1093/sysbio/syr087.

170. Wills MA (2001) How good is the fossil record of arthropods? An assessment using the stratigraphic congruence of cladograms. Geol J 36: 187–210. doi:10.1002/gj.882.

171. Warnock RCM, Yang Z, Donoghue PCJ (2011) Exploring uncertainty in the calibration of the molecular clock. Biol Lett 8: 156–159. doi:10.1098/rsbl.2011.0710.

172. Rehm P, Borner J, Meusemann K, von Reumont BM, Simon S, et al. (2011) Dating the arthropod tree based on large-scale transcriptome data. Mol Phylogenet Evol 61: 880–887. doi:10.1016/j.ympev.2011.09.003.

173. Wheat C, Wahlberg N (2013) Phylogenomic insights into the Cambrian Explosion, the colonization of land and the evolution of flight in Arthropoda. Syst Biol 62: 93–109.

174. R Developement Core Team (2011) R: A Language and Environment for Statistical Computing. Available: http://www.R-project.org.

175. Vas Z, Csorba G, Rózsa L (2012) Evolutionary co-variation of host and parasite diversity—the first test of Eichler’s rule using parasitic lice (Insecta: Phthiraptera). Parasitol Res 111: 393–401.

176. Durden L, Musser G (1994) The sucking lice (insecta Anoplura) of the world: a Taxonomic checklist with records of mammalian hosts and geograpahical distributions. Bull Am Museam Nat Hist 218: 1–90.

177. Cryan JR, Svenson GJ (2010) Family‐level relationships of the spittlebugs and froghoppers (Hemiptera: Cicadomorpha: Cercopoidea). Syst Entomol 35: 393–415. doi:10.1111/j.1365-3113.2009.00520.x.

178. Whiting MF, Whiting AS, Hastriter MW, Dittmar K (2008) A molecular phylogeny of fleas (Insecta: Siphonaptera): origins and host associations. Cladistics 24: 677–707. doi:10.1111/j.1096-0031.2008.00211.x.

179. Kathirithamby J (2009) Host-Parasitoid Associations in Strepsiptera. Annu Rev Entomol 54: 227–249. doi:10.1146/annurev.ento.54.110807.090525.

180. Zhang Z (2011) Animal biodiversity: An outline of higher-level classification and survey of taxonomic richness. Magnolia Press. 239 p.

181. Lienhard C, Smithers CN (2002) Psocoptera (Insecta): World Catalogue and Bibliography. Muséum d’histoire naturelle. 745 p.

182. Noyes JS (2003) Universal Chalcidoidea Database. World Wide Web electronic publication. Available: http://www.nhm.ac.uk/chalcidoids.

183. Ascher JS, Pickering J (2012) Discover life bee species guide and world checklist (Hymenoptera: Apoidea: Anthophila. Available: http://www.discoverlife.org/mp/20q?guide=Apoidea_species.

184. Pulawski W (2012) Catalog of Sphecidae sensu lato. Available: http://research.calacademy.org/ent/catalog_sphecidae.

185. Deem LS (2012) Dermaptera Species File. Version 1.0/4.1. [October 2012]. Available: http://Dermaptera.SpeciesFile.org.

186. Otte D, Spearman L, Stiewe M (2012) Mantodea Species File Online. Version 1.0/4.1. [October 2012]. Available: http://Mantodea.SpeciesFile.org.

187. Eades D (2012) Polyneoptera Species File Online. Version 1.0/4.1. [October 2012]. Available: http://Polyneoptera.SpeciesFile.org.

188. Penny N (1997) World Checklist of Extant Mecoptera Species. Available: http://research.calacademy.org/redirect?url=http://researcharchive.calacademy.org/research/entomology/Entomology_Resources/mecoptera/index.htm.

189. Maehr MD, Eades D (2012) Embioptera Species File Online. Version 1.0/4.1. [October 2012]. Available: http://Embioptera.SpeciesFile.org.

190. DeWalt RE, Neu-Becker U, Stueber G (2012) Plecoptera Species File Online. Version 1.0/4.1. [October 2012]. Available: http://Plecoptera.SpeciesFile.org.

191. Bourgoin T (2012) FLOW (Fulgoromorpha Lists on The Web): a world knowledge base dedicated to Fulgoromorpha. Version 8 [October 2012]. Available: http://hemiptera-databases.org/flow/.

192. Deitz L, Wallace MS, Dietrich CH, Mckamey SH, Rothschild MJ (2010) Treehoppers; Aetalionidae, Melizoderidae, and Membracidae (Hemiptera)[October 2012]. Available: http://treehoppers.insectmuseum.org/public/site/treehoppers/home.

193. Alfaro ME, Santini F, Brock C, Alamillo H, Dornburg A, et al. (2009) Nine exceptional radiations plus high turnover explain species diversity in jawed vertebrates. Proc Natl Acad Sci 106: 13410–13414. doi:10.1073/pnas.0811087106.

194. Brown JW, FitzJohn RG, Alfaro ME, Harmon LJ (2012) MEDUSA: Modeling Evolutionary Diversification Using Stepwise AIC,. Available: http://www.webpages.uidaho.edu/~lukeh/software/software.html. Accessed 24 February 2013.

195. Magallon S, Sanderson MJ (2001) Absolute Diversification Rates in Angiosperm Clades. Evolution 55: 1762–1780. doi:10.1111/j.0014-3820.2001.tb00826.x.

196. Harmon LJ, Weir JT, Brock CD, Glor RE, Challenger W (2008) GEIGER: investigating evolutionary radiations. Bioinformatics 24: 129–131. doi:10.1093/bioinformatics/btm538.

197. Mayhew PJ (2007) Why are there so many insect species? Perspectives from fossils and phylogenies. Biol Rev 82: 425–454. doi:10.1111/j.1469-185X.2007.00018.x.

198. Rabosky DL (2007) LASER: A Maximum Likelihood Toolkit for Detecting Temporal Shifts in Diversification Rates From Molecular Phylogenies. Evol Bioinforma Online 2: 247–250.

199. Davis RB, Baldauf SL, Mayhew PJ (2010) Many hexapod groups originated earlier and withstood extinction events better than previously realized: inferences from supertrees. Proc R Soc B Biol Sci 277: 1597 –1606. doi:10.1098/rspb.2009.2299.

200. Mayhew PJ (2002) Shifts in Hexapod Diversification and what Haldane could have said. Proc R Soc B Biol Sci 269: 969–974.

201. Mayhew PJ (2003) A tale of two analyses: estimating the consequences of shifts in hexapod diversification. Biol J Linn Soc 80: 23–36. doi:10.1046/j.1095-8312.2003.00217.x.

202. Stadler T (2011) Mammalian phylogeny reveals recent diversification rate shifts. Proc Natl Acad Sci 108: 6187–6192. doi:10.1073/pnas.1016876108.

203. Near TJ, Dornburg A, Eytan RI, Keck BP, Smith WL, et al. (2013) Phylogeny and tempo of diversification in the superradiation of spiny-rayed fishes. Proc Natl Acad Sci 110: 12738–12743. doi:10.1073/pnas.1304661110.

204. Beutel RG, Pohl H (2006) Endopterygote systematics – where do we stand and what is the goal (Hexapoda, Arthropoda)? Syst Entomol 31: 202–219. doi:10.1111/j.1365-3113.2006.00341.x.

205. Blanke A, Wipfler B, Letsch H, Koch M, Beckmann F, et al. (2012) Revival of Palaeoptera—head characters support a monophyletic origin of Odonata and Ephemeroptera (Insecta). Cladistics 28: 560–581. doi:10.1111/j.1096-0031.2012.00405.x.

206. Blanke A, Greve C, Wipfler B, Beutel RG, Holland BR, et al. (2013) The Identification of Concerted Convergence in Insect Heads Corroborates Palaeoptera. Syst Biol 62: 250–263. doi:10.1093/sysbio/sys091.

207. Thomas JA, Trueman J, Rambaut A, Welch JJ (2013) Relaxed phylogenetics and the Palaeoptera problem: Resolving deep ancestral splits in the insect phylogeny. Syst Biol 62: 285–297.

208. Kjer KM, Carle F, Litman J, Ware J (2006) A Molecular Phylogeny of Hexapoda. Arthropd Syst Phylogeny 64: 35–44.

209. Yoshizawa K, Johnson KP (2003) Phylogenetic position of Phthiraptera (Insecta: Paraneoptera) and elevated rate of evolution in mitochondrial 12S and 16S rDNA. Mol Phylogenet Evol 29: 102–114. doi:10.1016/S1055-7903(03)00073-3.

210. Lo N, Beninati T, Stone F, Walker J, Sacchi L (2007) Cockroaches that lack Blattabacterium endosymbionts: the phylogenetically divergent genus Nocticola. Biol Lett 3: 327–330. doi:10.1098/rsbl.2006.0614.

211. Ware JL, Litman J, Klass K-D, Spearman LA (2008) Relationships among the major lineages of Dictyoptera: the effect of outgroup selection on dictyopteran tree topology. Syst Entomol 33: 429–450. doi:10.1111/j.1365-3113.2008.00424.x.

212. Djernæs M, Klass K-D, Picker MD, Damgaard J (2012) Phylogeny of cockroaches (Insecta, Dictyoptera, Blattodea), with placement of aberrant taxa and exploration of out-group sampling. Syst Entomol 37: 65–83. doi:10.1111/j.1365-3113.2011.00598.x.

213. McKenna DD, Farrell BD (2010) 9-Genes reinforce the phylogeny of Holometabola and yield alternate views on the phylogenetic placement of Strepsiptera. PLoS ONE 5: e11887. doi:10.1371/journal.pone.0011887.

214. Longhorn SJ, Pohl HW, Vogler AP (2010) Ribosomal protein genes of holometabolan insects reject the Halteria, instead revealing a close affinity of Strepsiptera with Coleoptera. Mol Phylogenet Evol 55: 846–859. doi:10.1016/j.ympev.2010.03.024.

215. Sharkey MJ, Carpenter JM, Vilhelmsen L, Heraty J, Liljeblad J, et al. (2012) Phylogenetic relationships among superfamilies of Hymenoptera. Cladistics 28: 80–112. doi:10.1111/j.1096-0031.2011.00366.x.

216. Pilgrim EM, Von Dohlen CD, Pitts JP (2008) Molecular phylogenetics of Vespoidea indicate paraphyly of the superfamily and novel relationships of its component families and subfamilies. Zool Scr 37: 539–560. doi:10.1111/j.1463-6409.2008.00340.x.

217. Farrell BD (1998) “Inordinate fondness” explained: Why are there so many beetles? Science 281: 555 –559. doi:10.1126/science.281.5376.555.

218. Arnett RH, Thomas MC, Skelley PE, Frank JH (2010) American Beetles, Volume II: Polyphaga: Scarabaeoidea through Curculionoidea. CRC Press. 888 p.

219. Marshall S (2012) Flies: The Natural History and Diversity of Diptera. Firefly books.

220. Davis RB, Baldauf SL, Mayhew PJ (2009) Eusociality and the success of the termites: insights from a supertree of dictyopteran families. J Evol Biol 22: 1750–1761. doi:10.1111/j.1420-9101.2009.01789.x.
